# Tet-mediated DNA hydroxymethylation regulates retinal neurogenesis by modulating cell-extrinsic signaling pathways

**DOI:** 10.1371/journal.pgen.1006987

**Published:** 2017-09-19

**Authors:** Pawat Seritrakul, Jeffrey M. Gross

**Affiliations:** 1 Department of Molecular Biosciences and Institute for Cellular and Molecular Biology, The University of Texas at Austin, Austin, TX, United States of America; 2 Departments of Ophthalmology, and Developmental Biology, The Louis J. Fox Center for Vision Restoration, The McGowan Institute for Regenerative Medicine, The University of Pittsburgh School of Medicine, Pittsburgh, PA, United States of America; The Babraham Institute, UNITED KINGDOM

## Abstract

DNA hydroxymethylation has recently been shown to play critical roles in regulating gene expression and terminal differentiation events in a variety of developmental contexts. However, little is known about its function during eye development. Methylcytosine dioxygenases of the Tet family convert 5-methylcytosine (5mC) to 5-hydroxymethylcytosine (5hmC), an epigenetic mark thought to serve as a precursor for DNA demethylation and as a stable mark in neurons. Here, we report a requirement for Tet activity during zebrafish retinal neurogenesis. In *tet2*^*-/-*^*;tet3*^*-/-*^ mutants, retinal neurons are specified but most fail to terminally differentiate. While differentiation of the first born retinal neurons, the retinal ganglion cells (RGCs), is less affected in *tet2*^*-/-*^*;tet3*^*-/-*^ mutants than other retinal cell types, the majority of RGCs do not undergo terminal morphogenesis and form axons. Moreover, the few photoreceptors that differentiate in *tet2*^*-/-*^*;tet3*^*-/-*^ mutants fail to form outer segments, suggesting that Tet function is also required for terminal morphogenesis of differentiated retinal neurons. Mosaic analyses revealed a surprising cell non-autonomous requirement for tet2 and tet3 activity in facilitating retinal neurogenesis. Through a combination of candidate gene analysis, transcriptomics and pharmacological manipulations, we identified the Notch and Wnt pathways as cell-extrinsic pathways regulated by tet2 and tet3 activity during RGC differentiation and morphogenesis. Transcriptome analyses also revealed the ectopic expression of non-retinal genes in *tet2*^*-/-*^*;tet3*^*-/-*^ mutant retinae, and this correlated with locus-specific reduction in 5hmC. These data provide the first evidence that Tet-dependent regulation of 5hmC formation is critical for retinal neurogenesis, and highlight an additional layer of complexity in the progression from retinal progenitor cell to differentiated retinal neuron during development of the vertebrate retina.

## Author summary

Tet enzymes function to convert methylated cytosines (5mC) to hydroxymethylated cytosines (5hmC), an epigenetic mark associated with active transcription or as a precursor to demethylation. Here, we generated zebrafish *tet2*^*-/-*^*;tet3*^*-/-*^ mutants, which are deficient in the ability to convert 5mC to 5hmC. We identified functions for Tet enzymes in regulating gene expression and cell type-specific differentiation of retinal progenitor cells (RPCs) into neurons and glia during retinal development. Specifically, in *tet2*^*-/-*^*;tet3*^*-/-*^ mutants, despite relatively normal expression of specification markers, the majority of retinal cell types do not express markers of differentiation and they fail to undergo terminal differentiation and morphogenesis. Genome-wide expression profiling identified down-regulation of numerous retinal genes in *tet2*^*-/-*^*;tet3*^*-/-*^ mutants, and surprisingly, upregulation of cardiac and skeletal muscle-specific genes that are not normally expressed in the eye. Mechanistically, tet2 and tet3 function upstream of cell-extrinsic signaling pathways to enable specified RPCs to undergo terminal differentiation. This study is the first detailed analysis of Tet function during eye development and identifies an exciting new layer of epigenetic regulation operating during retinal neurogenesis.

## Introduction

In vertebrates, the majority of CpG sequences in the genome are characterized by addition of a methyl group to the 5th carbon of cytosine residues, 5mC [1]. Hypermethylation within promoters/enhancers is associated with reduced gene transcription [2], while gene body methylation directly correlates with expression [3]. Indeed, DNA methylation is critical for silencing imprinted genes and transposons [4–6]. Subsets of genes are differentially methylated according to tissue and cell type, and DNA methylation is thought to be a mechanism whereby cell type-specific expression patterns are set during terminal differentiation [7, 8], and by which some somatic progenitor cell populations are maintained [9–12].

Three main biochemical events orchestrate DNA methylation. First, *de novo* methylation, mediated by Dnmt3-family proteins, functions to methylate regions of hypomethylated DNA and is required for tissue-specific differentiation during development [8, 13–15]. Second, maintenance methylation, mediated by DNA methyltransferase-1 (Dnmt1), copies the methylation pattern from existing DNA strands on to nascent daughter strands during DNA replication, a process that is important for maintaining the identities of actively proliferating cell populations [10, 16]. Third, demethylation is the mechanism by which 5mC is removed from the genome. Far less is known about DNA demethylation but several biochemical pathways have been proposed to be involved and these include: replication-dependent passive dilution, direct base excision by the DNA repair machinery, and active enzymatic conversion of 5mC (reviewed in [17]). Of these pathways, most evidence supports the latter and a role for members of the ten-eleven translocation (Tet) family of methylcytosine dioxygenases. These enzymes mediate the conversion of 5mC to 5-hydroxymethylcytosine (5hmC), which can then be converted to non-methylated cytosine [18–20].

Recent studies have identified roles for Tet proteins and DNA hydroxymethylation during vertebrate development, stem cell maintenance and in diseases such as cancer. In mouse, *Tet1* and *Tet2* knockouts are viable and fertile, while *Tet1*^*-/-*^*;Tet2*^*-/-*^ double knockouts show a partially penetrant perinatal lethality associated with imprinting abnormalities [21]. A recently generated Tet1 knockout mouse showed forebrain defects at late gastrulation and high mortality [22]. *Tet1/2/3* triple knockout ES cells possess a massive loss of 5hmC, deregulated gene expression, and an impaired ability to differentiate [23]. In frog, depletion of Tet3 by a translation-blocking morpholino (MO) affects early neural development and causes an eyeless phenotype [24]. *tet2* MO-based knockdown in zebrafish results in mild defects in erythropoiesis [25]; however, mutations in *tet2* do not cause any overt embryonic phenotype, although *tet2*^*-/-*^ adults develop progressive age-related clonal myelodysplasia [26]. More recently, overlapping roles for *tet2* and *tet3* during hematopoietic stem cell differentiation have been identified [27]. Likely related to their functions during hematopoiesis, Tet proteins are also associated with a number of hematological malignancies in humans (reviewed in [28]).

Within the nervous system, Tet expression and 5hmC enrichment is detected in the developing mouse brain [29, 30]. 5hmC levels increase during neuronal differentiation, with enrichment at enhancers and also within gene bodies of neuronal genes [30]. This enrichment is interesting because it is not associated with subsequent demethylation, in agreement with a study that shows biochemical stability of 5hmC marks within the genome [31]. Beyond loss-of-function experiments, *Tet3* overexpression in mouse olfactory neurons results in an increase in 5hmC levels, altered gene expression, and defects in axon targeting [32], and tet3 activity is upregulated in dorsal root ganglia neurons during axon regeneration [33].

More recent work has shown that Tet proteins regulate both intrinsic and extrinsic pathways during development. Intrinsically, Tet activity is required during hematopoiesis [34] and B-cell development [35]. Tets also modulate the activity of extrinsic signaling pathways in a variety of contexts. Tets suppress Wnt pathway activity during early mouse development to balance mesoderm and neuroectoderm fates [36], while at later stages in the intestinal epithelium, Tet1 is required for Wnt pathway activation [37]. During gastrulation, Tet activity regulates the Nodal pathway by suppressing the expression of Nodal inhibitors [38].

Although DNA methylation and hydroxymethylation have been studied extensively in the context of stem cell programming and disease, far less is known about their roles during development and in regulating organogenesis to create a complex structure like the retina. The vertebrate retina consists of seven main cell types that perform distinct functions in phototransduction and visual signal transmission (reviewed in [39]). These cells are organized in the three retinal layers: the ganglion cell layer (GCL), outer nuclear layer (ONL), and inner nuclear layer (INL). The retina develops from a common pool of seemingly indistinguishable multipotent retinal progenitor cells (RPCs), which ultimately give rise to all retinal cells in a stereotyped time order (reviewed in [40]).

While much has been learned regarding the specification events that direct RPCs to distinct retinal cell fates, we know little about the epigenetic regulation of these events, or the mechanisms underlying terminal differentiation and morphogenesis of retinal neurons and glia. Indeed, no studies have determined whether Tet function or DNA hydroxymethylation are required during retinal development. To address this topic we functionally inactivated the *tet2* and *tet3* genes in zebrafish and identified defects in retinal development that resulted from deregulated gene expression in *tet2*^*-/-*^*;tet3*^*-/-*^ mutants. This is the first detailed analysis of Tet function during vertebrate retinal development, and our data support a model in which Tet-mediated regulation of 5hmC levels is critical for retinal neurogenesis.

## Results

### *tet2* and *tet3* expression and functional inactivation

Several studies have reported the expression of Tet-family genes in developing embryos and in tissues, including the eye (e.g. [24, 29]). Zebrafish possess three Tet-family genes (*tet1*, *tet2* and *tet3*), and these are orthologous to mouse and human Tet1-3 (S1A Fig). At 24 hours post fertilization (hpf) all three *tet* genes are expressed broadly throughout the embryo, including the eye (S1B–S1D Fig). At 48 and 72hpf, *tet1* expression is faint in the head and is expressed at comparatively lower levels in the eye than *tet2* and *tet3* (S1E and S1H Fig). At 48 and 72hpf, *tet2* and *tet3* are strongly expressed in the anterior of the embryo and, in the eye, transcripts are enriched in the GCL and INL, and are more faintly detected in the ONL (S1F, S1G, S1I and S1J Fig).

**Fig 1:**
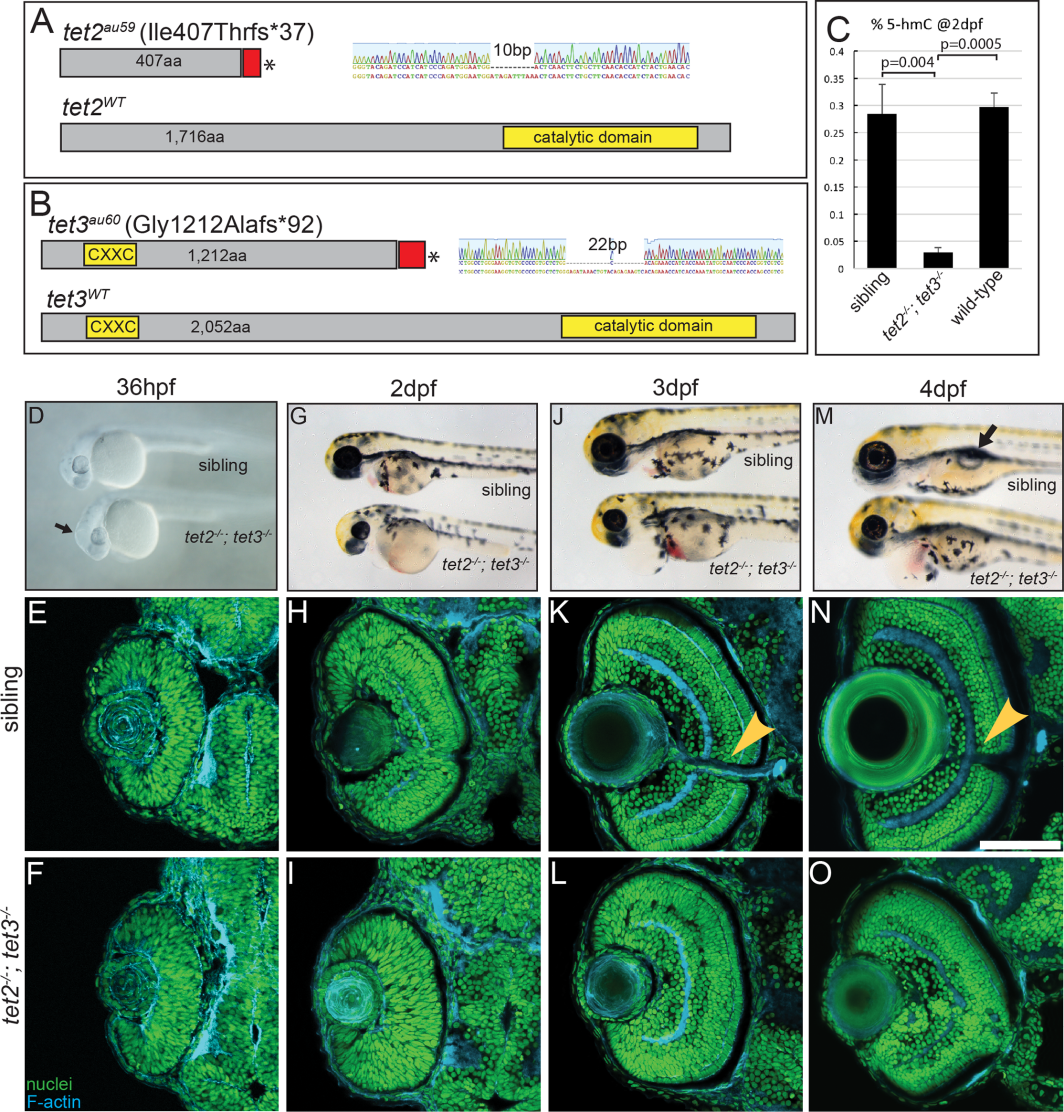
*tet2*^*-/-*^*;tet3*^*-/-*^ mutants are deficient in 5mC → 5hmC conversion and display abnormalities in retinal development. (A) *tet2*^*au59*^ mutants possess a 10bp deletion resulting in a frameshift, insertion of 37 incorrect aa’s (red), and a premature stop codon, truncating the protein at amino acid (aa) 444 of 1,716aa. (B) *tet3*^*au60*^ mutants possess a 22bp deletion, resulting in a frameshift, insertion of 92 incorrect aa’s (red), and a premature stop codon, truncating the protein at aa 1,304 of 2,052. (C) Genomic DNA isolated from 2dpf *tet2*^*-/-*^*;tet3*^*-/-*^ mutants shows a >9 fold reduction in global 5hmC levels when compared to phenotypically wild-type siblings and genetically wild-type embryos in an ELISA (n = 20 embryos, p = 0.004 and p = 0.0005, respectively; Error bars = ± 1 S.D.). (D) At 36hpf, *tet2*^*-/-*^*;tet3*^*-/-*^ mutants are identifiable based on a kinked head and slightly enlarged brain (arrow). (E,F) Retinae of 36hpf *tet2*^*-/-*^*;tet3*^*-/-*^ mutants are morphologically similar to wild-types. (G) At 2dpf, *tet2*^*-/-*^*;tet3*^*-/-*^ mutants are microphthalmic, possess cardiac edema and their heads are smaller than phenotypically wildtype siblings. (H-I) The retina is not laminated, with retinal cells appearing progenitor-like in morphology when compared to phenotypically wild-type siblings. (J) Cardiac edema becomes progressively enlarged at 3dpf in *tet2*^*-/-*^*; tet3*^*-/-*^ embryos. (K,L) The retina remains poorly laminated in *tet2*^*-/-*^*;tet3*^*-/-*^ mutants, and they lack a morphologically obvious optic nerve (arrowhead in sibling). (M) A 4dpf *tet2*^*-/-*^*; tet3*^*-/-*^ embryos do not possess an inflated swim bladder (arrow). (N,O) The retina remains poorly laminated and they lack a morphologically obvious optic nerve (arrowhead in sibling). DNA (green), F-actin (cyan). Dorsal is up and anterior to the left. Scale bar = 80μm.

Because *tet2* and *tet3* were the only Tet-family genes that showed prominent retinal expression past 24hpf, and a recent report demonstrated that *tet1* was dispensable for normal zebrafish development and for DNA hydroxymethylation [27], we focused on *tet2* and *tet3* for functional perturbations. Mutant alleles were created by designing transcription activator-like effector nucleases (TALENs) targeting the first exon of *tet2* and the sixth exon of *tet3* [41]. Injected mosaic founders were screened for germline transmission, and mutant alleles were detected and sorted by restriction fragment length polymorphisms (RFLP) and then sequenced (Fig 1A and 1B; S2 Fig). Two alleles were identified and maintained: *tet2*^*au59*^ mutants possess a 10bp deletion which is predicted to cause a frameshift beginning at amino acid (aa) 407, inserting 37 incorrect amino acids and truncating the protein at aa 444 out of 1,716 [c.1,219_1,229delATAGATTTAA, p.Ile407Thrfs*37], and *tet3*^*au60*^ mutants possess a 22bp deletion mutation, which is predicted to cause a frameshift beginning at aa 1,212, inserting 92 incorrect aa and truncating the tet3 protein at aa 1,304 out of 2,052 [c.4,860_4,881delGAGATAAACTGTACAGAGAAGT, p.Gly1,212Alafs*92].

*tet2*^*-/-*^ and *tet3*^*-/-*^ mutants develop normally with no visible phenotype and they are homozygous viable (S2 Fig). This is an unsurprising result given their close phylogenetic relationship and overlapping expression domains, and it is consistent with recent reports [26, 27]. Thus, *tet2*^*-/-*^*;tet3*^*-/-*^ mutants were generated by in-crossing double heterozygous adults (*tet2*^*+/-*^*;tet3*^*+/-*^). *tet2*^*-/-*^*;tet3*^*-/-*^ mutants were recovered at an expected Mendelian ratio (6.62%; n = 103 mutants/1,554 embryos). At 36hpf, *tet2*^*-/-*^*;tet3*^*-/-*^ mutants displayed a distinct morphological phenotype where the anterior portion of the brain was enlarged and kinked when compared to wild-type siblings (Fig 1D). At 2 days post-fertilization (dpf), *tet2*^*-/-*^*;tet3*^*-/-*^ mutants were microphthalmic, mildly hypopigmented, and displayed deformed craniofacial features, cardiac edema, and blood pooling, phenotypes that perdure through 4dpf (Fig 1G, 1J and 1M).

*tet2*^*au59*^ and *tet3*^*au60*^ mutations are predicted to truncate the proteins upstream of the C-terminal catalytic domain [20] and therefore encode null or severe loss of function alleles. At 2dpf and 5dpf, *tet2* transcripts were detectable in phenotypically wild-type siblings, but not in *tet2*^*-/-*^*; tet3*^*-/-*^ mutants, indicating that *tet2* transcripts are degraded through nonsense-mediated decay (NMD) (S3A Fig). *tet3* transcripts were still present in both sibling and *tet2*^*-/-*^*;tet3*^*-/-*^ mutants (S3B Fig). To assess tet3 protein, Western blots were performed on 3dpf phenotypically wild-type siblings and *tet2*^*-/-*^*;tet3*^*-/-*^ mutants (S3C and S3D Fig). tet3 protein was undetectable in *tet2*^*-/-*^*;tet3*^*-/-*^ mutants. These data indicate that *tet2*^*au59*^ and *tet3*^*au60*^ are null alleles. To experimentally validate the loss of catalytic function in *tet2*^*-/-*^*;tet3*^*-/-*^ mutants, we utilized a sensitive enzyme-linked immunosorbent assay (ELISA) to quantify whole embryo 5hmC levels. At 2dpf, genetically wild-type and phenotypically wild-type siblings possessed genome-wide 5hmC levels of 0.30% and 0.28%, respectively, consistent with published levels in various isolated mouse tissues [22, 42]. By comparison, *tet2*^*-/-*^*;tet3*^*-/-*^ mutants possessed a significant reduction in 5hmC levels (0.03%, p<0.005) indicating that *tet2*^*au59*^ and *tet3*^*au60*^ mutants lack almost all 5hmC in their genome (Fig 1C).

To analyze retinal development in *tet2*^*-/-*^*;tet3*^*-/-*^ mutants, we performed histology from 36hpf to 4dpf and assessed overall retinal structure. At 36hpf, no obvious differences between retinal morphology in *tet2*^*-/-*^*;tet3*^*-/-*^ mutants and wild-type siblings were evident (Fig 1E and 1F). At 2dpf, *tet2*^*-/-*^*;tet3*^*-/-*^ mutant retinae appeared progenitor-like in morphology, as little evidence of lamination or neuronal differentiation was evident (Fig 1H and 1I). At 3dpf, *tet2*^*-/-*^*;tet3*^*-/-*^ mutant retinae displayed some evidence of lamination, though this was reduced when compared to that in phenotypically wild-type siblings. Moreover, while cells populated the inner retina, and a GCL and INL were discernable, all *tet2*^*-/-*^*;tet3*^*-/-*^ mutants lacked a morphologically obvious optic nerve (Fig 1K and 1L). Retinal defects remained prevalent at 4dpf with *tet2*^*-/-*^*;tet3*^*-/-*^ mutant retinae continuing to possess lamination defects and no apparent formation of an optic nerve, despite the presence of cells in the inner retinal area normally occupied by RGCs (Fig 1N and 1O).

### Cell cycle dynamics in early progenitor cells are disrupted in *tet2*^*-/-*^*;tet3*^*-/-*^ mutants

**Fig 2:**
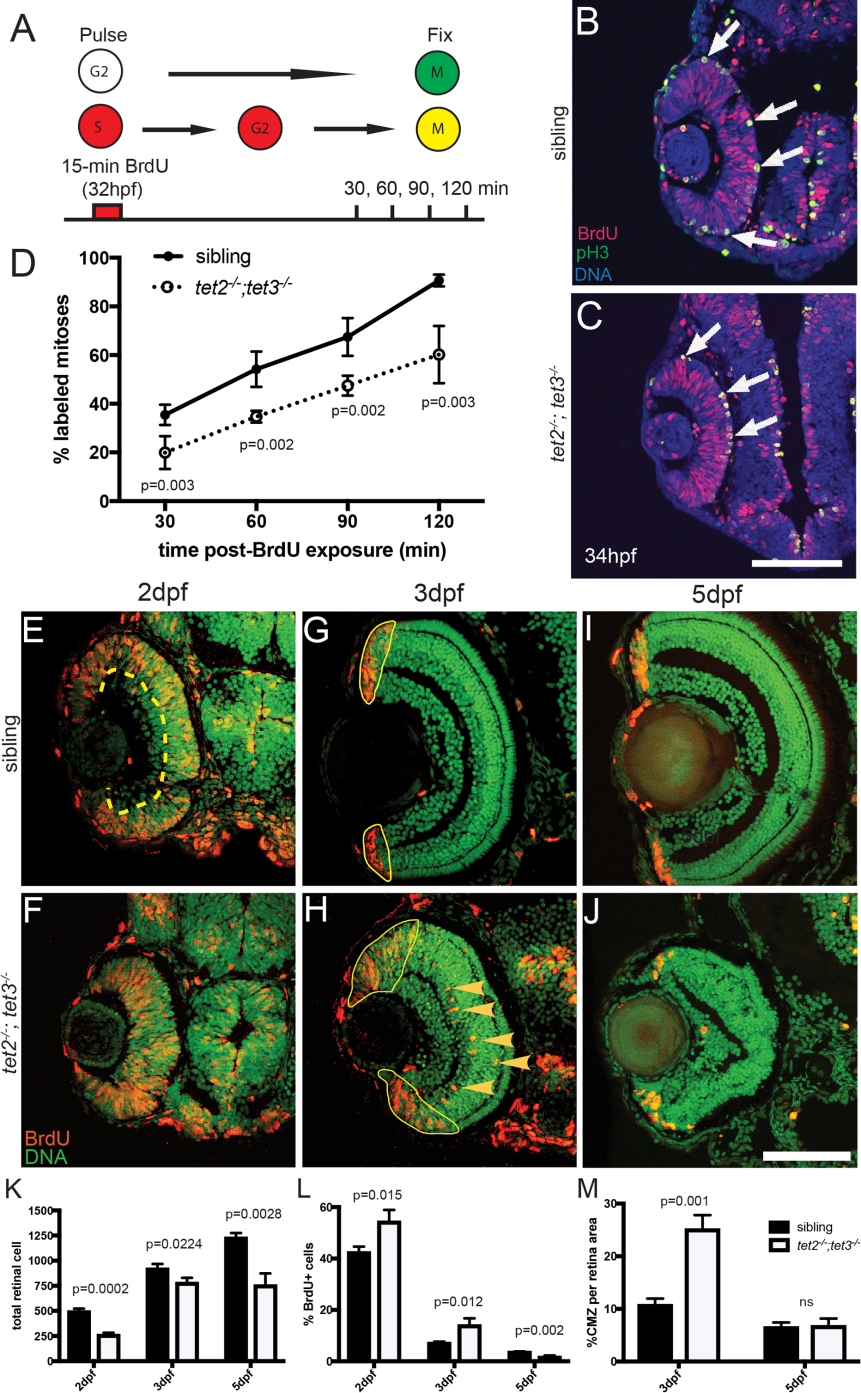
RPC cell cycle dynamics are disrupted in *tet2*^*-/-*^*;tet3*^*-/-*^. A) Percent labeled mitoses (PLM) assay was performed by treating embryos for 15 minutes in bromodeoxyuridine (BrdU) pulse at 32hpf, rinsed, fixed at 30, 60, 90, and 120 minutes post-treatment for immunostaining. (B,C) Cells in S-phase during BrdU pulse (BrdU^+^; red) and cells in G2/M-phase at fixation (pH3^+^; green) were counted. Cells that were proliferative during the BrdU pulse and then undergo mitosis are double positive (BrdU^+,^pH3^+^; yellow; arrows). (D) *tet2*^*-/-*^*;tet3*^*-/-*^ retinae show significantly lower proportion of labeled mitotic events ([BrdU^+^pH3^+^]/pH3^+^) at all four time points. By the end of 120-minute window, nearly all ‘labeled’ RPCs completed S to M phase transition in wild-type siblings, compared to only 50% in *tet2*^*-/-*^*;tet3*^*-/-*^ mutants (n = 5 embryos per condition per timepoint analyzed). (E-M) At later time points, BrdU incorporation assays over 2-hour time windows revealed that retinal progenitor cells remain proliferative longer and proliferate ectopically in *tet2*^*-/-*^*;tet3*^*-/-*^ mutants. (E,F) At 2dpf, cells within the central retina of wild-type sibling embryos (dotted line) are no longer proliferative, correlating with cell cycle exit and differentiation. (G,H) At 3dpf, the only proliferative cells in the wild-type retina are located in the CMZ (outlined). In *tet2*^*-/-*^*;tet3*^*-/-*^ mutants, this proliferative region is significantly expanded. Ectopically proliferating cells are also observed outside of the CMZ at significantly higher numbers than wild-type siblings (arrowheads). (I,J) At 5dpf, both sibling and *tet2*^*-/-*^*;tet3*^*-/-*^ eyes possess proliferative CMZs. (K) Total retinal cell count per section is significantly lower in *tet2*^*-/-*^*;tet3*^*-/-*^ mutants compared to siblings at all time points, correlating with microphthalmia. (L) *tet2*^*-/-*^*;tet3*^*-/-*^ mutant retinae possess a significantly higher percentage of proliferative (BrdU^+^) cells at 2 and 3dpf, but lower at 5dpf. (M) Percentage of CMZ area per total retina is significantly higher in *tet2*^*-/-*^*;tet3*^*-/-*^ mutant at 3dpf, but not significantly different at 5dpf. N = 3 embryos per condition per time point for E-M. Dorsal is up and anterior to the left in all images. All error bars = ± 1 S.D. All p-values calculated using two-tailed, unpaired t-test. Scale bar = 50μm in B-C; 80μm in E-J.

Because retinal cells in *tet2*^*-/-*^*;tet3*^*-/-*^ mutants appeared to be progenitor-like in morphology at 2dpf, and lamination defects persisted at 3dpf and 4dpf, a phenotype associated with elongated proliferation and/or defects in cell cycle exit in zebrafish [43–45], we next determined cell cycle dynamics in *tet2*^*-/-*^*;tet3*^*-/-*^ mutant retinal cells. First we assayed the S to M phase progression by utilizing the percent labeled mitoses (PLM) assay [43, 46, 47]. Embryos were treated with a 15-minute bromodeoxyuridine (BrdU) pulse at 32hpf, washed, fixed at 30, 60, 90, and 120 minutes post-treatment, and immunostained for BrdU and phosphohistone H3 (pH3) (Fig 2A). Cells in S-phase during the BrdU pulse (BrdU^+^) and cells in late G2/M-phase at the time of fixation (pH3^+^) were quantified. Cells that were proliferative during the BrdU pulse and then undergo mitosis are double positive (BrdU^+^,pH3^+^). In contrast, cells that were not in S-phase during the BrdU pulse, but still undergo mitosis, are only pH3^+^. Thus, the proportion of these cells {(BrdU^+^pH3^+^)/pH3^+^} represent the ‘labeled’ mitotic events. In *tet2*^*-/-*^*;tet3*^*-/-*^ mutants, the percent labeled mitoses are significantly lower than sibling at all time points examined (Fig 2B–2D). Nearly 100% of ‘labeled’ RPCs in wild-type embryos completed the S to M phase transition by the end of 120-minute time window, while only 50% of *tet2*^*-/-*^*;tet3*^*-/-*^ mutants competed this transition. This indicates that between 32-34hpf, *tet2*^*-/-*^*;tet3*^*-/-*^ RPCs are progressing from S to M at a slower rate than wildtype RPCs.

**Fig 3:**
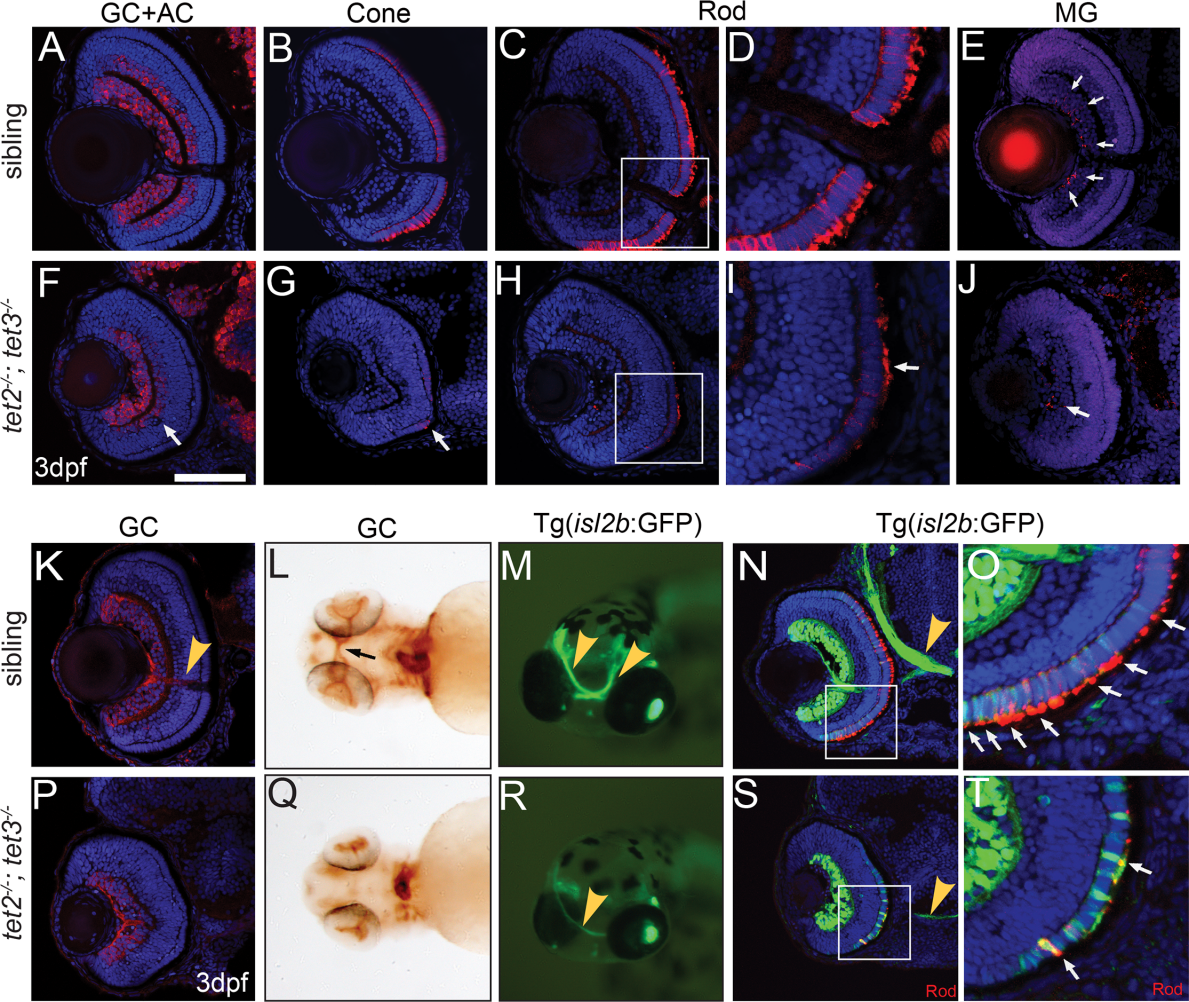
*tet2*^*-/-*^*;tet3*^*-/-*^ retinal cells do not undergo terminal differentiation. (A,F) HuC/D labels RGCs and amacrine cells (ACs), which are reduced in number and located only in the central region of the INL in *tet2*^*-/-*^*;tet3*^*-/-*^ retinae (arrow). (B,G) *tet2*^*-/-*^*;tet3*^*-/-*^ mutant retinae almost entirely lack zpr-1^+^ red/green cones (arrow); (C,D,H,I) possess few zpr-3^+^ rods (arrow), and of those that are zpr-3^+^, outer segments are severely attenuated or almost absent (arrow). (E,J) *tet2*^*-/-*^*;tet3*^*-/-*^ retinae also possess few zrf-1^+^ Müller glia (arrows in wild-type). In all cases, marker^+^ cells are located in the central/ventral part of the retina. (K,P) Zn8 detects neurolin, a protein enriched on RGCs and the optic nerve (arrowhead in K). (L,Q) Zn8 staining reveals the optic nerve in the choroid fissure and optic chiasm (arrow) of wild-type embryos but not in *tet2*^*-/-*^*;tet3*^*-/-*^ mutants. (M-N) *isl2b*:GFP transgenics express GFP in RGCs and PRs, clearly labeling the optic nerve in whole-mount and section views (arrowhead). (R,S) The *tet2*^*-/-*^*;tet3*^*-/-*^ optic nerve is very thin, often unilaterally formed, but, when present, correctly routed to the brain. (O,T) The *isl2b*:GFP signal overlaps zpr-3 (rod) marker in the cell body and outer segments in siblings (arrows). In *tet2*^*-/-*^*;tet3*^*-/-*^, few *isl2b*:GFP^+^ cells are zpr-3^+^ (arrows), further suggesting that specified cells are not terminally differentiated. Few outer segments have also formed in *tet2*^*-/-*^*;tet3*^*-/-*^ mutants. DNA (blue), antibody stain (red). All images are 3dpf. n>5 for each marker. Dorsal is up and anterior to the left. Scale bar = 80μm in A-K, P.

At later time points, BrdU incorporation assays over 2-hour pulse windows showed that, although *tet2*^*-/-*^*;tet3*^*-/-*^ mutant eyes were smaller and contained fewer cells (Fig 2E–2K), they contained a significantly higher percentage of BrdU^+^ cells at both 2dpf and 3dpf (Fig 2L). At 2dpf, central retinal cells of wildtype embryos have exited the cell cycle and differentiated, while in *tet2*^*-/-*^*;tet3*^*-/-*^ mutant eyes the central retina remained proliferative (Fig 2E and 2F). By 3dpf, cells of the wildtype retina have exited the cell cycle and differentiated except for those in the ciliary marginal zone (CMZ) at the retinal periphery which remains proliferative throughout the life of the animal (Fig 2G and 2H) [48]. In *tet2*^*-/-*^*;tet3*^*-/-*^ mutants, most cells within the central retina were no longer BrdU^+^, but the peripheral (CMZ) domain was significantly expanded (Fig 2M). Moreover, ectopic proliferative cells were detected in the inner retina, outside of the *tet2*^*-/-*^*;tet3*^*-/-*^ CMZ (Fig 2H), a phenotype not observed in wildtype siblings. The identity of these cells remains unclear, although we have ruled out the possibility of these being Müller glia (MG) cells, as they do not co-stain with the MG-specific marker, zrf-1/gfap (Fig 3J). By 5dpf, a distinct zone of proliferation is present in *tet2*^*-/-*^*;tet3*^*-/-*^ mutants, indicating that the mutant RPCs eventually completed cell cycle exit, and that ectopically proliferative cells are no longer present (Fig 2I and 2J).

While the PLM and BrdU incorporation results suggest that elongation of the cell cycle during early retinal development underlies microphthalmia in *tet2*^*-/-*^*;tet3*^*-/-*^ mutants, apoptosis of RPCs or newly differentiated neurons could also contribute. To determine whether apoptosis plays a role in *tet2*^*-/-*^*;tet3*^*-/-*^ mutant retinal defects, we performed terminal deoxynucleotidyl transferase dUTP nick-end labeling (TUNEL) assays. No increase in apoptotic cells was detected in *tet2*^*-/-*^*;tet3*^*-/-*^ mutant until after 3dpf (S4 Fig), indicating that apoptosis is unlikely to account for microphthalmia in *tet2*^*-/-*^*;tet3*^*-/-*^ mutants.

### Retinal cell differentiation is impaired in *tet2*^*-/-*^*;tet3*^*-/-*^ embryos

Given the impaired retinal lamination and cell cycle progression defects in *tet2*^*-/-*^*;tet3*^*-/-*^ mutants, we next asked whether *tet2*^*-/-*^*;tet3*^*-/-*^ retinal cells differentiate into neurons and Müller glia. In wild-type embryos, all retinal neuron and glial cell types are differentiated by 72hpf [i.e. [43, 49, 50]]. An early neuronal marker, HuC/D, detects RGCs and amacrine cells (ACs) [51, 52]; in *tet2*^*-/-*^*;tet3*^*-/-*^ mutants, while HuC/D^+^ cells were detected in the GCL and INL, their number in the INL was significantly lower and the few HuC/D^+^ cells present were restricted to the central-most region of the retina (Fig 3A and 3F; S5 Fig). *tet2*^*-/-*^*;tet3*^*-/-*^ mutants almost entirely lacked zpr1-positive red/green double cones (Fig 3B and 3G), zpr-3-positive rods (Fig 3C, 3D, 3H and 3I), and zrf-1/gfap-positive Müller glia (Fig 3E and 3J). *tet2*^*-/-*^*;tet3*^*-/-*^ mutants possess positive immunoreactivity to Zn8/neurolin, which detects differentiated RGCs [53], although the region of Zn8^+^ RGCs does not extend as far to the retinal periphery as in wild-type siblings (Fig 3K and 3P). For each of the markers tested, immunoreactive cells were restricted to the central-most retina and none were detected more peripherally, corresponding with the expanded zone of proliferation detected at 3dpf in BrdU assays (Fig 2).

In sections of *tet2*^*-/-*^*;tet3*^*-/-*^ mutant eyes, no optic nerve was detected (Figs 1–3). It was formally possible that an optic nerve was present, but that axons were misrouted inside of the retina, a phenotype associated with defects in axonal pathfinding [54]. To exclude this possibility, we performed whole-mount chromogenic labeling using the Zn8/neurolin antibody to label RGC axons [54]. In 3dpf phenotypically wild-type siblings, Zn8 labeled intra-retinal RGC axons that extend along the vitreal surface of the eye to generate the optic nerve, as well as the optic nerve itself as it passes through the choroid fissure (CF) and into the optic chiasm (Fig 3L). In *tet2*^*-/-*^*;tet3*^*-/-*^ mutants, Zn8 was only detected within the GCL and in a few axons within the choroid fissure (Fig 3Q). As a more sensitive optic nerve labeling assay, we utilized a transgenic reporter, *Tg(isl2b*:*GFP)*^*zc7*^, which expresses GFP in RGCs, and a subset of developing photoreceptors (PRs) [55]. Sibling embryos possessed a strong GFP signal in RGC cell bodies and axons along the entire length of the optic nerve, clearly visible in both section and whole-mount preparations (Fig 3M–3O). In *tet2*^*-/-*^*;tet3*^*-/-*^ mutants, while 12% of 41 mutant embryos lacked an optic nerve entirely, the remainder possessed an optic nerve, formed either bilaterally (39%) or unilaterally (49%); however, in these embryos, the optic nerve was thin and appeared to be composed of very few axons (Fig 3M and 3R). For the few axons that did leave the eye, retinotectal projections appeared to be normal, even in embryos with unilateral optic nerves (Fig 3R). Taken together, these data indicate that the terminal differentiation and morphogenesis of RGCs is affected in *tet2*^*-/-*^*;tet3*^*-/-*^ mutants, and not axon pathfinding.

Photoreceptors (PRs) undergo terminal differentiation and morphogenesis to form outer segments rich in photoreceptive molecules for phototransduction; this starts in the ventro-nasal patch and spreads through the retina (Fig 3B–3D) [50, 56, 57]. Sporadic rods and cones are detected in the central region of the retina in *tet2*^*-/-*^*;tet3*^*-/-*^ mutants, likely the earliest born PRs, but none are detected more peripherally (Fig 3G–3I). Moreover, in sibling embryos, while outer segments are well formed by 72hpf and highly immuoreactive to zpr-1 (arrestin3a) and zpr-3 (rhodopsin), the few PRs that differentiate in *tet2*^*-/-*^*;tet3*^*-/-*^ mutants possess little to no outer segment material (Fig 3D and 3I). This is further highlighted by staining *isl2b*:GFP embryos with zpr-3. Only a few *isl2b*:GFP^+^ PRs express zpr-3 and of those that do, they have nearly undetectable outer segments (Fig 3O and 3T). Given the dramatic reductions in optic nerve size and in PR outer segment formation, we conclude that RGCs and PRs of *tet2*^*-/-*^*;tet3*^*-/-*^ mutants, despite expressing some markers of terminal differentiation, do not complete morphogenesis.

### Neuronal specification occurs normally in *tet2*^*-/-*^*;tet3*^*-/-*^ mutants

**Fig 4:**
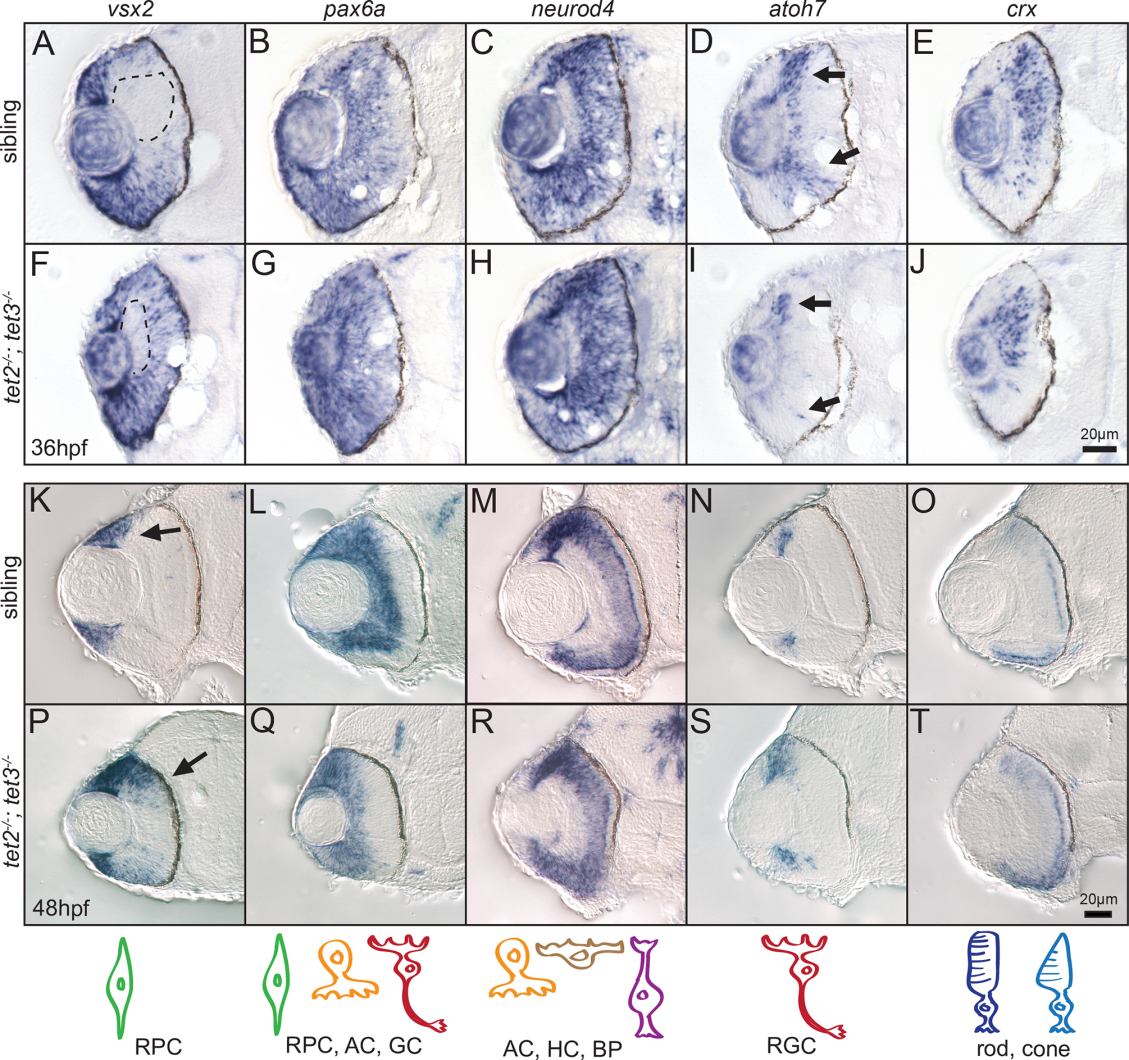
Expression of retinal cell fate specification markers is largely normal in *tet2*^*-/-*^*;tet3*^*-/-*^ embryos. Expression of genes involved in retinal neurogenesis was detected by in situ hybridization at 36hpf and 48hpf. (A,F) At 36hpf, *vsx2* is expressed in the retinal progenitor cells (RPCs) and turned off as they differentiate, first in the central retina (A, dotted area). This zone of differentiation is still present in *tet2*^*-/-*^*;tet3*^*-/-*^ mutant (F, dotted area). (K,P) At 48hpf, expression of *vsx2* is slightly expanded in *tet2*^*-/-*^*;tet3*^*-/-*^ mutants (K,P arrows) but otherwise appears in a normal pattern. (B,C,G,H,L,M,Q,R) Expression of *pax6a* (marker for RPC, amacrine, and ganglion cells) and *neuroD4* (marker for amacrine, horizontal, and bipolar cells) is relatively normal in *tet2*^*-/-*^*;tet3*^*-/-*^ mutants when compared to sibling embryos at both 36hpf and 48hpf. (D,I,N,S) Ganglion cell precursors express *atoh7* as they exit the RPC pool, become specified and start undergoing differentiation. *atoh7* expression is present in the correct location in *tet2*^*-/-*^*;tet3*^*-/-*^ mutants, compared to sibling embryos at both 36hpf and 48hpf. (E,J,O,T) *crx* is expressed in cells fated to become photoreceptors (rods and cones) and this expression pattern appears relatively normal in *tet2*^*-/-*^*;tet3*^*-/-*^ mutants. Scale bar = 20μm. n>8 per gene for each time point. Drawings on bottom row represent cell type detected in each corresponding column.

To begin to determine the molecular mechanism responsible for the reduction of terminally differentiated retinal neurons in *tet2*^*-/-*^*;tet3*^*-/-*^ mutants, we next asked if neuronal specification factors were properly expressed (Fig 4). At 36hpf, *vsx2* is expressed in proliferative RPCs, and turned off as these cells exit the cell cycle and begin to differentiate (Fig 4A, dotted area). In *tet2*^*-/-*^*;tet3*^*-/-*^ mutants, this zone of differentiation is noticeably smaller (Fig 4F). At 48hpf, *vsx2* expression is localized in proliferative cells at the periphery of the retina (Fig 4K) [58]. This zone of *vsx2* expression was expanded in *tet2*^*-/-*^*;tet3*^*-/-*^ mutants when compared to wild-type siblings (Fig 4P), corresponding to the expanded zone of proliferation observed in the BrdU labeling assay (Fig 2).

*pax6a* is normally expressed in RPCs, in addition to RGCs and ACs [59]; *neurod4* is expressed in ACs, horizontal and bipolar cells [60]; *atoh7* is expressed in the committed precursors undergoing specification to become RGCs [52], and *crx* is expressed in specified PRs [61] (Fig 4B–4E and 4L–4O). Despite terminal differentiation defects observed in retinae of *tet2*^*-/-*^*;tet3*^*-/-*^ mutants, they retain relatively normal spatial and temporal expression of specification markers at 36hpf and 48hpf (Fig 4G–4J and 4Q–4T, respectively), suggesting that RPC specification is unaffected during retinal neurogenesis in the absence of tet2 and tet3 function.

### tet2 and tet3 regulate cell non-autonomous effects during retinal neuron differentiation

**Fig 5:**
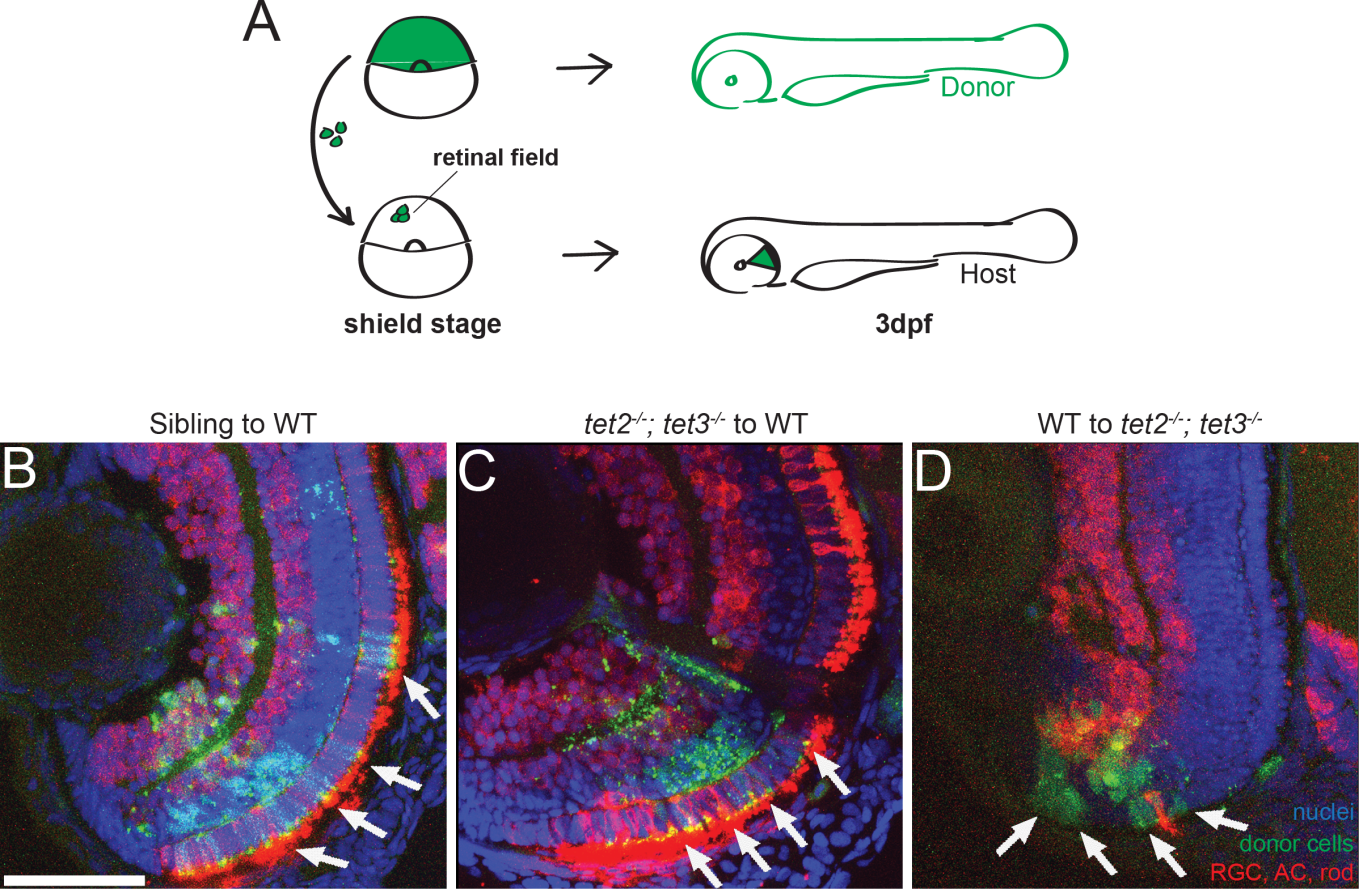
Tet2 and tet3 regulate retinal neurogenesis cell non-autonomously. (A) Chimeric embryos were generated by blastomere transplantation from fluorescent dextran-labeled donor into unlabeled host. Retinae composed of clones from wild-type and *tet2*^*-/-*^*;tet3*^*-/-*^ mutant cells were analyzed at 3dpf. (B) Sibling donor to wildtype host transplants yielded clones of donor cells (green; arrows) that differentiated normally into retinal neurons including AC, RGC, rods (red), and display proper lamination (n = 5). (C) Similarly, *tet2*^*-/-*^*;tet3*^*-/-*^ mutant cells transplanted into genetically wildtype hosts also differentiated normally, and the regions of the retina containing mutant clones were also properly laminated (n = 3). (D). Genetically wild-type donor cells transplanted into *tet2*^*-/-*^*;tet3*^*-/-*^ mutant host retinae failed to undergo neurogenesis and remained undifferentiated (green, arrows) (n = 3). DNA (blue), HuC/D + zpr-3 antibody stain (red), transplanted donor clones (green). All images are 3dpf. Dorsal is up and anterior to the left. Scale bar = 50μm

Tet proteins are known to regulate both intrinsic and extrinsic pathways during development and differentiation events in a range of tissues. For example, tet activity is required intrinsically during hematopoiesis [34] and B-cell differentiation [35]. Recent studies have shown that Tet activity also modulates extrinsic pathways during development. In mouse embryos and embryonic stem cells, Tets function to negatively regulate Wnt pathway activity to balance mesoderm vs neuroectoderm fate choices [36], and during mouse gastrulation, they modulate Nodal pathway activity by controlling the expression of Lefty1, a nodal inhibitor [38]. Retinal cell type specification and differentiation depend on a multitude of intrinsic and extrinsic pathways [reviewed in [39, 62]], and given that Tet activity can modulate both intrinsic and extrinsic pathways during development, we next sought to determine whether tet2 and tet3 activities were required cell autonomously or cell non-autonomously during retinal neurogenesis. Chimeric embryos were generated by blastomere transplantation [63] to generate embryos whose retinae were composed of clones of wild-type and *tet2*^*-/-*^*;tet3*^*-/-*^ mutant cells. Donor embryos were injected with fluorescent dextran and clones were transplanted from labeled donors into unlabeled hosts at shield stage, targeting specifically to the retinal field where cells later develop into the retina [64]. At 3dpf, host embryos were analyzed through a combination of HuC/D and zpr-3 staining, to detect differentiated RGCs, ACs and/or rods (Fig 5A). Sibling to wild-type transplants yielded clones of cells that differentiated normally (Fig 5B). *tet2*^*-/-*^*;tet3*^*-/-*^ mutant cells transplanted into genetically wildtype hosts also differentiated normally into retinal neurons including RGCs, ACs, and rods, and the regions of the retina containing mutant clones were also properly laminated (Fig 5C). Genetically wildtype cells transplanted into *tet2*^*-/-*^*;tet3*^*-/-*^ mutant host retinae failed to undergo neurogenesis and remained undifferentiated (Fig 5D). Thus, the wild-type retina was able to support normal neurogenesis of *tet2*^*-/-*^*;tet3*^*-/-*^ mutant cells, while wild-type cells in a *tet2*^*-/-*^*;tet3*^*-/-*^ mutant retina did not differentiate properly. Taken together, these data demonstrate that tet2 and tet3 activity regulates retinal neuron differentiation via cell non-autonomous pathways, potentially by modulating the expression or activity of cell-extrinsic signaling molecules.

### The Notch and Wnt pathways function downstream of tet2 and tet3

**Fig 6:**
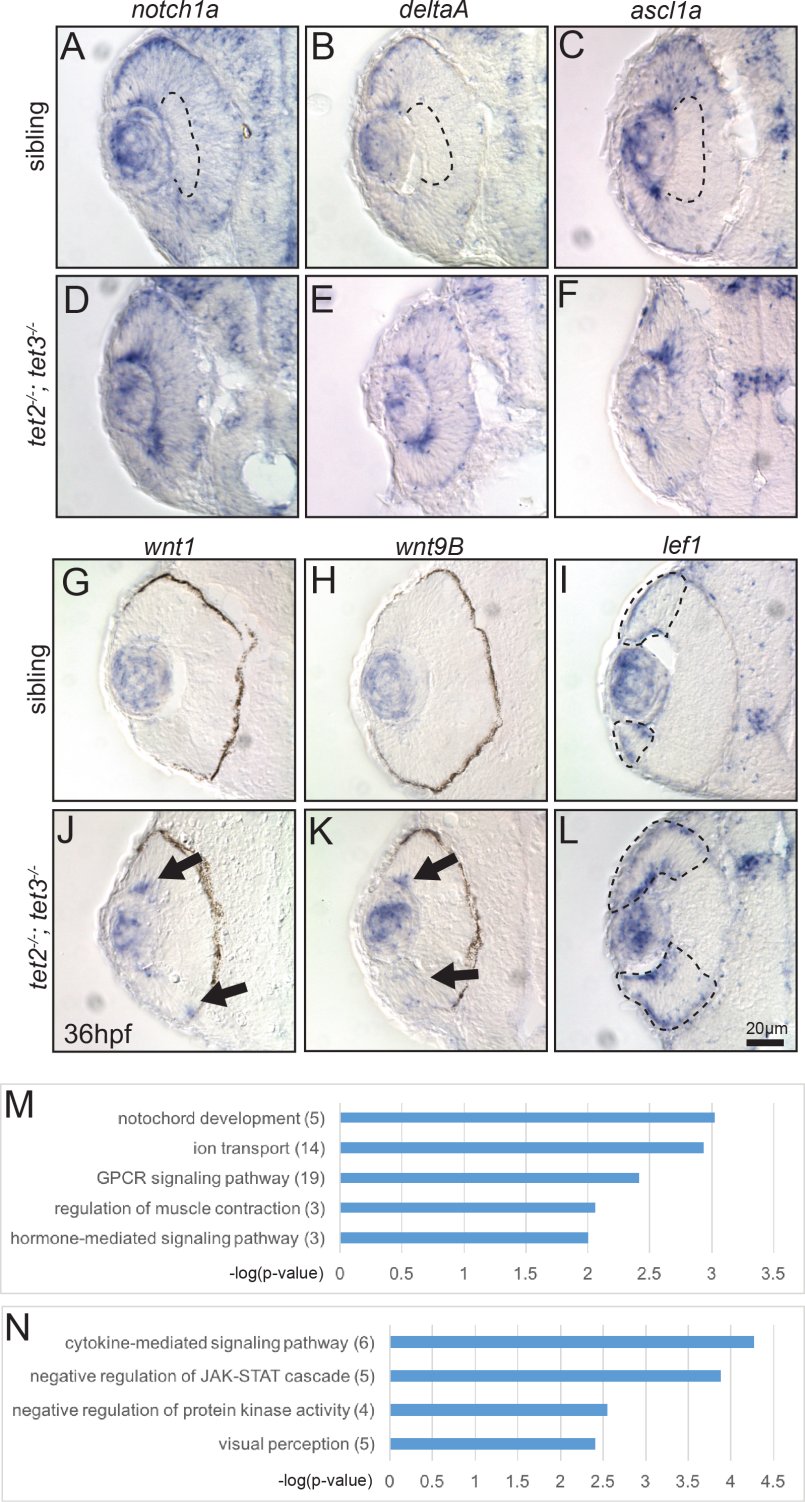
Gene expression is altered in *tet2*^*-/-*^*;tet3*^*-/-*^ mutants at 36hpf and differentially expressed genes include those encoding components of the Notch and Wnt pathways. (A-C) In sibling embryos at 36hpf, transcripts encoding components of Notch pathway (*notch1a*, *deltaA*, and *ascl1a*) are expressed in the eye but excluded from the inner part of central retina where cells have exited the cell cycle and differentiated (dotted area). (D-F) In *tet2*^*-/-*^*;tet3*^*-/-*^ mutants, these genes are expressed throughout the retina without a clear ‘zone’ of differentiation (n>8). (G,H,J,K) The expression domains of *wnt1* and *wnt9B*, are expanded in *tet2*^*-/-*^*;tet3*^*-/-*^ mutants (arrows; n = 7 for *wnt1*, n = 8 for *wnt9b*), consistent with RNA-Seq data. (I,L) Similarly, *lef1*, a downstream readout of Wnt pathway activity, is normally expressed in the peripheral edge of the retina, and this zone of expression is expanded in *tet2*^*-/-*^*;tet3*^*-/-*^ mutants (dotted areas; n>8). (M-N) Gene ontology (GO) analysis for biological pathways was performed using DAVID. Numbers in parentheses indicate number of genes enriched in each pathway. P-value cutoff = 0.01.

Because tet2 and tet3 appear to regulate retinal cell differentiation in a cell non-autonomous fashion, we next sought to identify potential factors responsible for these effects, utilizing both a candidate gene approach and an unbiased transcriptomic analysis. The Notch and Wnt pathways regulate retinal neurogenesis in mouse, zebrafish and Xenopus [65–68]; upregulation of these pathways prevents neuronal differentiation in a variety of contexts and resultant phenotypes resemble those in *tet2*^*-/-*^*;tet3*^*-/-*^ mutants [45, 68–70]. Thus, to determine whether the expression of Notch and Wnt pathway components, or the overall activities of the pathways, are regulated by tet2 and tet3, we first performed in situ hybridizations using probes specific to candidate genes in each pathway. In wild-type sibling embryos at 36hpf, *notch1a*, *deltaA*, and *ascl1a* are expressed in the eye but excluded from the inner part of central retina where RGCs, ACs and PRs are differentiating (Fig 6A–6C). However, in *tet2*^*-/-*^*;tet3*^*-/-*^ mutants, these genes are expressed uniformly throughout the retina, without a clear ‘zone’ of differentiation (Fig 6D–6F). Similarly, *lef1*, a downstream readout of the Wnt pathway [71], is expressed in the peripheral part of the retina of wild-type embryos at 36hpf (Fig 6I), and this zone of expression is expanded in *tet2*^*-/-*^*;tet3*^*-/-*^ mutants (Fig 6L). These expression patterns suggest that the Notch and Wnt pathways could have elevated activity in the *tet2*^*-/-*^*;tet3*^*-/-*^ mutant retina.

To complement these candidate gene studies, we performed RNA-seq using dissected *tet2*^*-/-*^*;tet3*^*-/-*^ mutant and phenotypically wildtype sibling eyes at 36hpf. Approximately 450 million reads were generated and mapped to GRCz10 [72, 73] at 85.1% mapping efficiency. In *tet2*^*-/-*^*;tet3*^*-/-*^ mutants, 278 genes were downregulated and 489 genes were upregulated (log_2_ fold-change above 2) as compared to wild-type siblings (S1 Table). Gene ontology (GO) analysis for biological pathways categorized many of the differentially expressed genes in the development of the visual perception, GPCR and cytokine-mediated signaling pathways, and ion transport (Fig 6M and 6N). Notably, the highest upregulated gene was *wnt9b* (log_2_ fold-change = 9.89), and many other members of the Wnt family were also upregulated (*wnt1*, *wnt3*, *wnt11r*, *wnt10a*) in *tet2*^*-/-*^*;tet3*^*-/-*^ mutant eyes (S1 Table). In situ hybridization using antisense probes for *wnt1* and *wnt9b* revealed that at 36hpf, while both genes showed faint expression at the peripheral edge of the retina in sibling embryos (Fig 6G and 6H, n = 7 for *wnt1*, n = 10 for *wnt9b*), in *tet2*^*-/-*^*;tet3*^*-/-*^ mutants, both genes were expressed in expanded domains (Fig 6J and 6K, n = 7 for *wnt1*, n = 8 for *wnt9b*), and at higher relative intensities, consistent with the RNA-seq data. When combined with mosaic analyses, these data support a model in which extrinsic signals, likely including Notch and Wnt-related pathways, are regulated by tet2 and tet3 activity during retinal development and in their absence, these pathways are overactive and impair terminal differentiation of retinal neurons.

**Fig 7:**
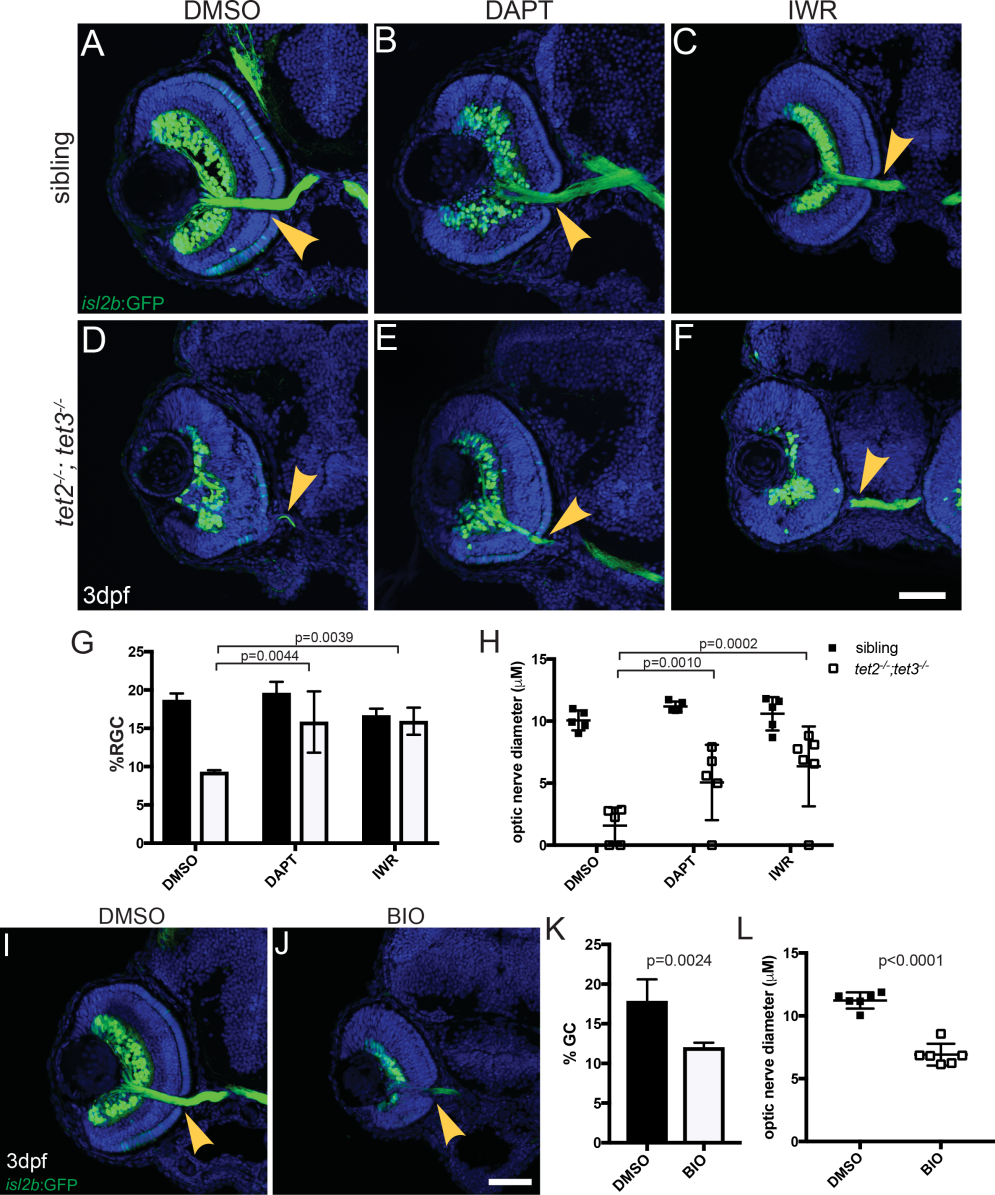
Tet2 and Tet3 function upstream of the Notch and Wnt pathways during RGC differentiation and morphogenesis. *tet2*^*-/-*^*;tet3*^*-/-*^ mutant and sibling embryos carrying *isl2b*:GFP were exposed to 50μM DAPT, 5μM IWR-1-endo, or 1% DMSO, during neurogenesis (24hpf to 72hpf) and analyzed for *isl2b*:GFP^+^ RGCs and axons. (A-C, G-H) No significant increases in optic nerve diameter or percentage of *isl2b*:GFP^+^ RGC per total cells (%RGC) were detected in sibling embryos treated with either 1% DMSO (vehicle), DAPT (Notch inhibitor), or IWR (Wnt inhibitor). (E,G,H) *tet2*^*-/-*^*;tet3*^*-/-*^ mutants treated with 50μM DAPT showed a significant increase in both the percentage of *isl2b*:GFP^+^ RGCs per retina and optic nerve diameter, when compared to DMSO-treated controls. (F-H) *tet2*^*-/-*^*;tet3*^*-/-*^ embryos treated with 5μM IWR also showed a significant increase in the percentage of *isl2b*:GFP^+^ RGCs per retina and optic nerve diameter. (I-L) Wnt signaling was upregulated by exposing wild-type embryos from 24hpf to 72hpf with 2μM BIO, a GSK3β inhibitor. (I,J) BIO-treated wild-type embryos showed reduced lamination, (K) a decreased percentage of *isl2b*:GFP^+^ RGCs per retina, and (L) decreased optic nerve diameter relative to DMSO-treated controls, (p = 0.00236 and p<0.0001). All error bars = ± 1 S.D.; n = 5 embryos (A-H) and n = 6 embryos (I-L) per condition analyzed; P-values calculated using two-way ANOVA with multiple comparisons (for G-H) and two-tailed, unpaired t-test (for K-L). Scale bar = 50μm.

To further test this model, we utilized pharmacological manipulations to determine if blocking Notch or Wnt activity could restore retinal neuron differentiation and morphogenesis in *tet2*^*-/-*^*;tet3*^*-/-*^ mutants. DAPT is a γ–secretase inhibitor that blocks the proteolytic cleavage of the intracellular domain of Notch, thus blocking its downstream signaling [74], and has been used extensively in zebrafish [66, 67, 74, 75]. Inhibitor of Wnt Response (IWR-1-endo) stabilizes Axin2 and promotes β-catenin degradation, effectively inhibiting Wnt signaling [76]. We exposed *tet2*^*-/-*^*;tet3*^*-/-*^ mutant and sibling embryos carrying the *isl2b*:GFP reporter to 50μM DAPT or 5μM IWR from the onset of neurogenesis (24hpf) until fixation at 3dpf. Interestingly, *tet2*^*-/-*^*;tet3*^*-/-*^ mutants treated with DAPT showed a significant increase in both the percentage of *isl2b*:GFP^+^ RGCs in the retina, and in optic nerve diameter, when compared to DMSO-treated controls (p = 0.0044 and p = 0.0010, respectively, 2-way ANOVA, Fig 7A–7H). No significant increases were detected in wild-type sibling embryos treated with either vehicle or DAPT (Fig 7G and 7H). Similarly, *tet2*^*-/-*^*;tet3*^*-/-*^ embryos treated with IWR also showed a significant increase in percentage of *isl2b*:GFP^+^ RGCs and optic nerve diameter (p = 0.0039 and p = 0.0002, respectively, 2-way ANOVA, Fig 7A–7H). Neither treatment rescued expression of *isl2b*:GFP^+^ PRs however, suggesting that other extrinsic pathways are likely regulated by tet2 and tet3 activity. These data support a model in which overactive Notch and/or Wnt signaling are partially responsible for neuronal differentiation/morphogenesis defects in *tet2*^*-/-*^*;tet3*^*-/-*^ mutants. To further test this model, we upregulated Wnt signaling by treating wild-type embryos from 24hpf to 3dpf with 2μM BIO, a GSK3β inhibitor [71, 77]. BIO-treated embryos showed dramatically reduced lamination, and decreases in the percentage of *isl2b*:GFP^+^ RGCs, and in optic nerve diameter relative to DMSO-treated controls, phenotypes that recapitulate those in *tet2*^*-/-*^*;tet3*^*-/-*^ mutants (p = 0.0024 and p<0.0001, Fig 7I–7L). Taken together, these data demonstrate that Wnt and Notch signaling pathways act downstream of tet2 and tet3 activity to regulate RGC differentiation and morphogenesis during zebrafish retinal neurogenesis.

### Gene expression and 5hmC distribution is disrupted in *tet2*^*-/-*^*;tet3*^*-/-*^ mutants

**Fig 8:**
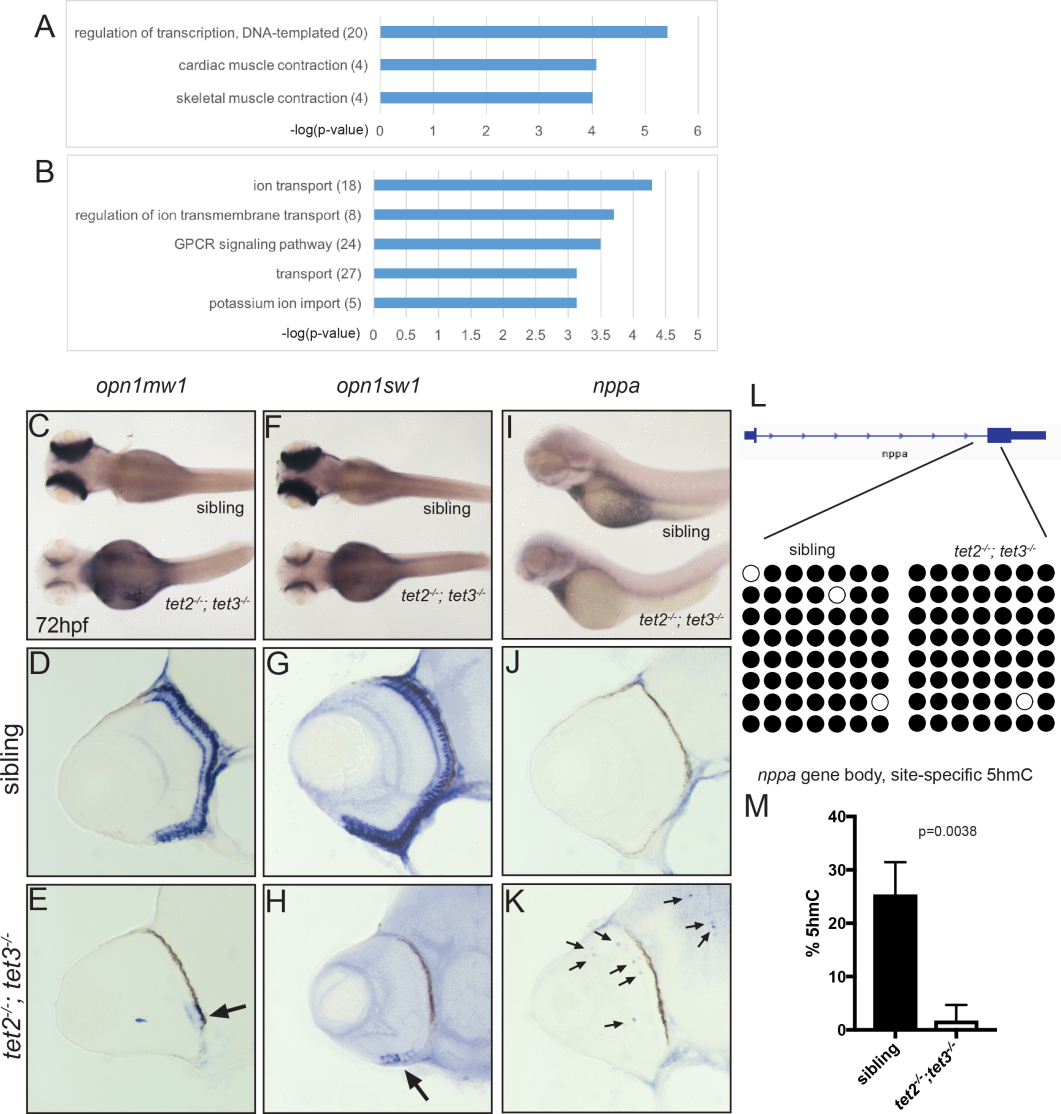
Gene expression and 5hmC levels are abnormal in *tet2*^*-/-*^*;tet3*^*-/-*^ mutant eyes at 72hpf. (A-B) GO analysis for biological pathways was performed using DAVID. Numbers in parentheses indicate number of genes enriched in each GO term. P-value cutoff = 0.001. In situ hybridization of (C-E) medium-wave sensitive (*opn1mw1*) and (F-H) short-wave sensitive opsin (*opn1sw1*). Transcripts of both genes are only detected in a few cells of the ventral retina in *tet2*^*-/-*^*;tet3*^*-/-*^ embryos (arrows in E,H; n>8). *natriuretic peptide a* (*nppa*) is normally expressed in the heart (I), and is not detected in the retina of wild-type embryos (J; n>8). (K) In *tet2*^*-/-*^*;tet3*^*-/-*^ embryos, ectopic *nppa* expressing cells were detected throughout the retina and brain (arrows) of all embryos examined (n = 16/16). (L) Bisulfite sequencing did not identify any changes in DNA methylation in any of the sixteen RNA-seq-identified target loci examined (S4 Table), including the *nppa* gene body. Bisulfite reads covering part of the first intron and second exon of *nppa* gene body are shown as black (methylated) or white (unmethylated). (M) Site-specific 5hmC quantification detected a significant, 20-fold reduction in 5hmC levels in the *nppa* gene body of 72hpf *tet2*^*-/-*^*;tet3*^*-/-*^ embryonic eye tissue, when compared to phenotypically wild-type siblings (p = 0.0038; two-tailed, unpaired t-test).

Despite early specification markers being expressed relatively normally during neurogenesis at 36 and 48hpf (Fig 4), retinal neurons still do not terminally differentiate. Thus, we next sought to determine the gene expression signatures of retinal cells at 72hpf as a means to infer their identities, and we again utilized RNA-Seq to assess these. Similar to 36hpf assays, total RNA was extracted from dissected 72hpf *tet2*^*-/-*^*;tet3*^*-/-*^ eyes and used for RNA-seq. Sixty-six million reads were generated and mapped to GRCz10 at 88.7% mapping efficiency. Comparisons of wild-type to *tet2*^*-/-*^*;tet3*^*-/-*^ mutants resulted in the identification of 212 upregulated genes and 451 downregulated genes that passed a threshold of at least a 2 log_2_ fold-change (Fig 8A and 8B; S2 Table). GO analysis was performed and, as expected, downregulated genes in *tet2*^*-/-*^*;tet3*^*-/-*^ mutants are members of pathways tightly linked to differentiated PRs; i.e. GPCR signaling, membrane transport and ion transport. Indeed, the most downregulated genes were those encoding proteins required for PR function such as opsins (e.g. *opn1mw1*, *opn1sw1*) and components of the visual cycle (e.g. *guca1c*, *guca1g*, *gnat2*, *grk1b*) (S2 Table), an unsurprising result given the near absence of terminally differentiated PRs in *tet2*^*-/-*^*;tet3*^*-/-*^ mutants (Fig 3). While the highest number of upregulated genes constituted the regulation of transcription category, other upregulated GO terms included skeletal muscle contraction and cardiac muscle contraction. These GO categories included genes that are not normally expressed in the eye; these included *nppa*, *vmhcl*, *chrng*, and *ucp1*, each of which encodes a protein involved in heart and muscle development and/or function (Fig 8B; S1 Table).

To verify RNA-seq results, we performed in situ hybridization using probes specific to selected differentially expressed genes. Both *opn1mw1* and *opn1sw1* were expressed in PRs at 72hpf of wild-type sibling embryos, and expression was largely absent in *tet2*^*-/-*^*;tet3*^*-/-*^ mutants, except in a small patch of cells in the ventral retina that corresponds to the region where differentiated PRs are detected (Fig 8C–8H). *nppa* (*natriuretic peptide precursor a*) was the most highly upregulated gene in *tet2*^*-/-*^*;tet3*^*-/-*^ mutant eyes (log_2_ fold-change = 6.3). *nppa* encodes the precursor to a peptide required for cardiovascular function and is normally only expressed in the embryonic heart [78]. All wild-type and *tet2*^*-/-*^*;tet3*^*-/-*^ mutant embryos showed normal heart expression of *nppa* (Fig 8I). Interestingly, however, in *tet2*^*-/-*^*;tet3*^*-/-*^ mutants *nppa* was ectopically expressed in the retina and brain of all embryos (n = 16/16 embryos), while no *nppa* expression was detected in the retina or brain of wild-type embryos (n = 0/7 embryos) (Fig 8I–8K).

To gain molecular insight into the epigenetic regulation of the differentially expressed genes in *tet2*^*-/-*^*;tet3*^*-/-*^ embryos and whether these expression changes correlated with changes in 5mC or 5hmC deposition, we performed locus-specific methylation analyses, using bisulfite conversion followed by sequencing for 5mC + 5hmC, and a glucosylation-digestion assay for 5hmC specifically. We targeted regions surrounding the transcription start sites (TSSs) of *opn1sw1*, *opn1mw1*, a CpG island near a gene cluster that contains multiple opsins, including *opn1mw1*, as well as the TSS and gene body of *nppa* (S3 Table). Out of eleven targets selected for bisulfite sequencing, we observed no difference in methylation status at any CpG sites. In both wild-type and *tet2*^*-/-*^*;tet3*^*-/-*^ mutants, all TSSs and the *nppa* gene body were fully methylated, and the opsin cluster CpG island was fully unmethylated (Fig 8L and S4 Table).

Bisulfite sequencing, while providing a nucleotide-resolution view of the methylation status of each CpG analyzed, is incapable of detecting differences between 5mC and 5hmC, meaning any CpG that appeared methylated in bisulfite assays could be either 5mC or 5hmC, or a mixture of both. To distinguish between these two epigenetic marks, we utilized glucosylation-digestion-based (Quest) assay to probe the presence of 5hmC at a glucosyl-sensitive restriction site, MspI. We selected targets that were located within or adjacent to the bisulfite-probed regions, due to the relatively sparse occurrence of MspI sites. Out of nine sites selected for analysis, only one, located in the gene body of *nppa*, showed a significant difference in 5hmC levels (S4 Table). Interestingly, 5hmC was reduced to a nearly undetectable level at this site in *tet2*^*-/-*^*;tet3*^*-/-*^ mutants, when compared to WT siblings where ~25% of the residues were hydroxymethylated (Fig 8M). These data demonstrate that *tet2* and *tet3* mutations result in defects in 5mC to 5hmC conversion within the *nppa* gene body, which could contribute to misregulated expression of the locus.

## Discussion

DNA hydroxymethylation and demethylation remain somewhat enigmatic processes in the field of epigenetics, with Tet protein function having only been identified recently (reviewed in [20]). Tet proteins are the main drivers of 5mC to 5hmC conversion and thereby key regulators of DNA demethylation [79]. However, in recent years, it has also become evident that they play roles in tissue-specific regulation of gene expression during development [24, 25, 27, 32, 34]. Despite these studies, we still know little about their developmental functions, and we know virtually nothing about Tet function during eye development.

**Fig 9:**
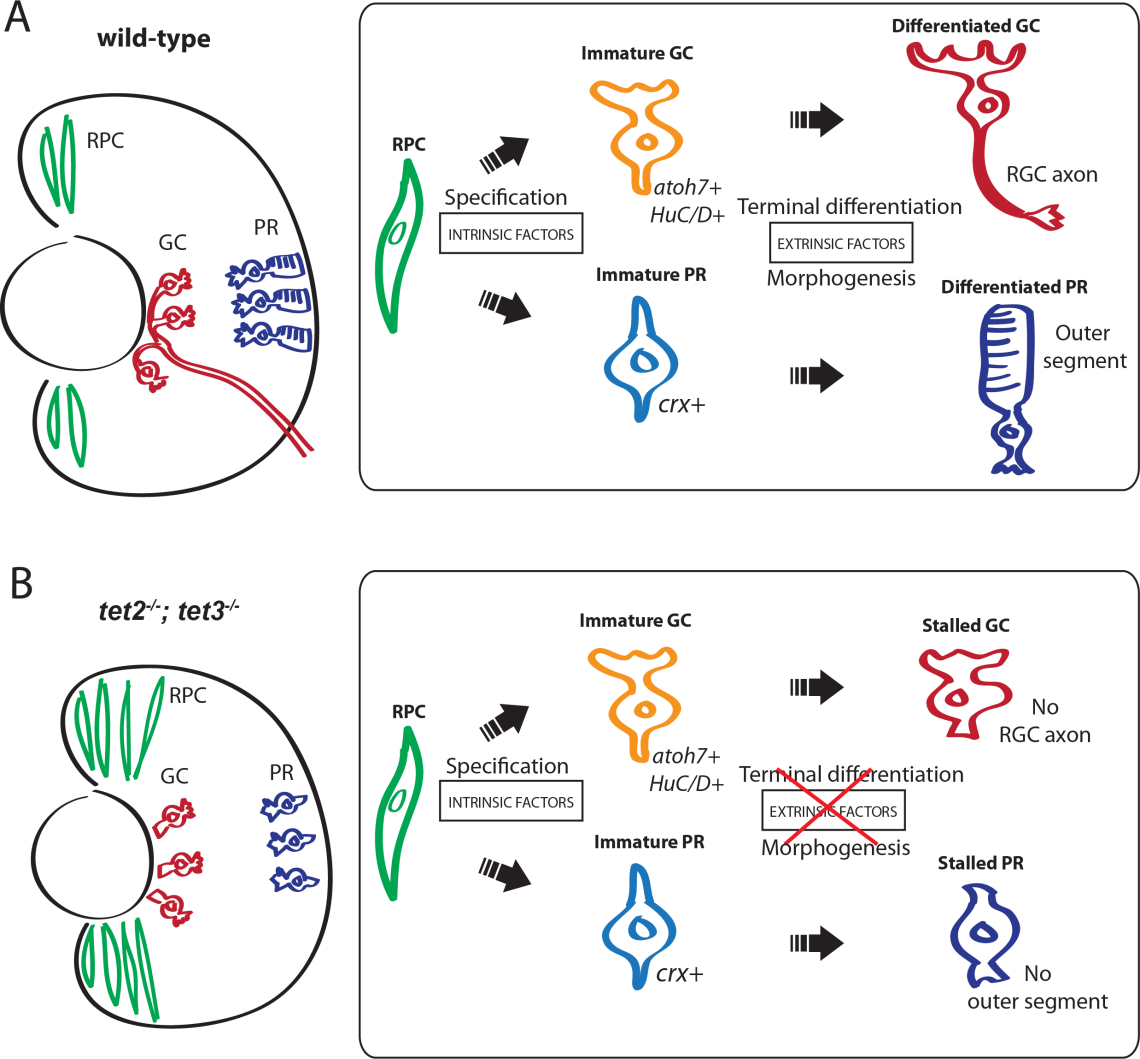
Schematic of Tet function during zebrafish retinal development. (A) During normal development, Tet proteins mediate epigenetic programming in retinal progenitor cells (RPCs; green) as they are specified into retinal neurons: retinal ganglion cells (RGCs, red) and photoreceptors (PRs, blue). These neurons undergo terminal differentiation and morphogenesis, generating axons that bundle into an optic nerve (for RGCs) and outer segments (for PRs). (B) In *tet2*^*-/-*^*;tet3*^*-/-*^ embryos, the RPC population is transiently expanded at early stages, but normal by 5dpf. Retinal neurons, despite being properly specified, fail to undergo terminal differentiation, and of those RGCs and PRs that do differentiate, many fail to undergo morphogenesis. These defects are likely caused by misregulation of cell-extrinsic factors required for terminal differentiation.

Here, we demonstrate that tet2 and tet3 play a critical role during development of the zebrafish retina. Our data indicate that tet2 and tet3 function redundantly in zebrafish to generate 5hmC, consistent with a recent report [27]. We demonstrate that tet2 and tet3 are critical regulators of retinal cell differentiation and morphogenesis, and that they act during early retinal development by modulating cell non-autonomous pathways. Loss of tet2 and tet3 function resulted in specific defects in retinogenesis, where the RPC population was transiently expanded. Despite relatively normal specification events, retinal cells failed to differentiate and, in the case of RGCs and PRs, failed to undergo terminal morphogenesis. These defects also correlated with mis-regulated gene expression and locus-specific defects in 5hmC formation in subsets of retinal cells at later stages of development. Based on these results, we propose a model wherein Tet proteins function to regulate gene expression during the differentiation of retinal cell types. In the absence of tet2 and tet3 function, gene expression is misregulated and differentiation and terminal morphogenesis of retinal neurons is perturbed (Fig 9).

Similar to the cellular differentiation defects identified here in *tet2*^*-/-*^*;tet3*^*-/-*^ retinae, loss of Tet2 and/or Tet3 function results in differentiation defects in the hematopoietic system of zebrafish [25, 27] and humans [34]. During hematopoiesis, Tet proteins turn on genes involved in erythropoiesis by hydroxymethylating and/or demethylating the their promoters, thereby enabling expression and ultimately, triggering differentiation. Conversely, it is well established that Tet1 is a critical player in stem cell maintenance where it functions to inhibit differentiation potential [80, 81], and loss of Tet1 impairs ESC self-renewal [18]. Therefore, Tet proteins play distinct roles in different contexts: they stimulate differentiation in tissue specific contexts (e.g. retinal, blood cells), and suppress differentiation in stem cells.

In *tet2*^*-/-*^*;tet3*^*-/-*^ mutants, early-born retinal cell types, RGCs and ACs, were less affected than later-born ones (cones, rods and Müller glia), which were almost completely absent. RGCs in *tet2*^*-/-*^*;tet3*^*-/-*^ mutants were *isl2b*:GFP^+^ and expressed Zn8, a marker of terminal differentiation [53]. However, Zn8^+^ RGCs were restricted to the central retina, and in a subset of mutants, no optic nerve (ON) was present, while in the remainder, a severely attenuated ON formed. In these latter embryos, we speculate that the RGCs generating axons are most likely the ‘pioneer’ axons [55] that originate from the few early-born RGCs that undergo terminal differentiation and morphogenesis, while the majority of RGCs fail to complete morphogenesis to form an axon. ACs were detected in fewer number and located in an even more limited zone within the central retina of *tet2*^*-/-*^*;tet3*^*-/-*^ mutants, and the few differentiated red/green double cones, rods or Müller glia detected in *tet2*^*-/-*^*;tet3*^*-/-*^ mutant retinae were always located in the central retina. Specification and differentiation in the zebrafish retina initiates in the ventronasal patch, adjacent to the optic nerve, and proceeds in a central to peripheral gradient [82]. That differentiated cells in *tet2*^*-/-*^*;tet3*^*-/-*^ mutants reside in these retinal regions strongly suggests that they represent the first born cells of each retinal cell type. Zebrafish embryos are also endowed with a maternally-derived supply of mRNA and protein (reviewed in [83]). Therefore, it is possible that the centrally located and partially differentiated early born cell types in *tet2*^*-/-*^*;tet3*^*-/-*^ mutants reflect a “maternal rescue”, and that defects in later born cell types result from depletion of maternally supplied tet2 and/or tet3. However, *tet* transcripts are not maternally deposited in zebrafish [29], and our 36hpf RNA-Seq analysis from *tet2*^*-/-*^*;tet3*^*-/-*^ mutants detects no expression of wild-type (maternally derived) *tet2* or *tet3* transcripts, making this scenario unlikely.

Alternatively, tet activity could become progressively more important in RPCs as they transition from producing early born cell types to later born ones, and as the specified cells undergo terminal differentiation and morphogenesis. Moreover, that the first born neurons of each class appeared to partially differentiate, tet2 and tet3 activity could become more important over time in each class of retinal neuron, where the first born neurons of the class develop independent of tet2 and tet3 function, while subsequent neurons require it. None-the-less, in this scenario, early born cells (and cell types) still require tet activity for terminal differentiation, because the majority of RGCs in *tet2*^*-/-*^*;tet3*^*-/-*^ mutants, though properly specified, do not form axons, and similarly in PRs, centrally-located cells are specified (*crx*^*+*^) and begin to differentiate (*isl2b*:GFP^+^, zpr-1^+^ or zpr-3^+^), but do not complete outer segment morphogenesis. These data suggest that while RGCs and early born PRs may be refractory to the absence of tet activity during the earliest phases of differentiation, tet activity is still required for terminal morphogenesis. Our speculation that epigenetic regulation plays an important role in terminal differentiation of retinal neurons is also supported by recent evidence in mouse where disruption of Dnmt1, 3a and 3b resulted in severe retinal defects in which some PRs were present, but they appeared disorganized and failed to form outer segments [84], defects reminiscent to those in *tet2*^*-/-*^*;tet3*^*-/-*^ mutants.

Mosaic analyses reveal that *tet2*^*-/-*^*;tet3*^*-/-*^ retinal phenotype occurs cell non-autonomously, and thus, that the effects of tet2 and tet3 loss of function during early retinal development are mediated by cell extrinsic events. Through a combination of candidate gene assays, transcriptomics and pharmacological manipulations, we demonstrate that elevated Notch and Wnt pathway activity is partially responsible for defects in retinal neurogenesis in *tet2*^*-/-*^*;tet3*^*-/-*^ mutants. However, because blocking these pathways only provided partial rescue of retinal defects in *tet2*^*-/-*^*;tet3*^*-/-*^ mutants, other signaling pathways are likely to be involved and modulated by tet activity during early retinal development. Hedgehog-PKA, TGFβ/BMP, and FGF are all cell-extrinsic pathways known to contribute to retinal neurogenesis, making these attractive candidates [85–87]. While surprising, these cell non-autonomous results are consistent with those from several other recently published studies on Tet function. Indeed, Tet activity was demonstrated to modulate Nodal activity during mouse gastrulation by intrinsically regulating the methylation status and expression of the Nodal inhibitors, Lefty1 and Lefty2 [38]. Tet activity has also recently been shown to modulate Wnt ligands or Wnt target gene activity in several contexts, either directly or indirectly [36, 37]. Perhaps the most interesting of these recent studies showed that in mouse ESCs and early embryos, Tet activity is required to balance neuroectoderm vs. mesoderm fates and to inhibit Wnt signaling [36]. In Tet1/2/3 deficient ES cells and embryos, neural cell fates were lost and instead, mesodermal fates like cardiac muscle were detected. These fate changes correlated with increased promoter methylation and decreased expression of the Wnt inhibitor, Sfrp4 and hyperactive Wnt pathway activity. This parallels what we observe in the *tet2*^*-/-*^*;tet3*^*-/-*^ mutant zebrafish retina, and is exciting, because it suggests that Tet-mediated modulation of the Wnt pathway, and possibly other cell-extrinsic signaling pathways, may be a conserved function for Tets during embryonic development and organogenesis. Finally, Tet function may also influence chromatin accessibility at the genomic regions surrounding Notch and Wnt genes, enabling other transcriptional regulators to access these loci. Tets have been shown to function in regulating local chromatin environments [30, 88–90].

From RNA-seq analysis, we detected a suite of cardiac and muscle genes ectopically expressed in the retinae of 72hpf *tet2*^*-/-*^*;tet3*^*-/-*^ mutants. Further analysis of one of these, *nppa*, revealed ectopic expression in the brain and eyes of *tet2*^*-/-*^*;tet3*^*-/-*^ mutants. This expression change correlated with an almost complete loss of gene body 5hmC deposition at the *nppa* locus. Locus-specific effects like this indicate that tet2 and tet3 may also function intrinsically during retinal development, in addition to modulating cell extrinsic pathways. Tet-mediated formation of 5hmC serves as a precursor to demethylation, or as an activating mark in its own right [30]. Tet-mediated 5hmC formation could play a direct role in silencing ectopic gene expression for genes like *nppa* during retinal development. Gene body 5mC methylation positively correlates with expression [3]; therefore, in this scenario, Tet-mediated conversion to 5hmC likely serves as a precursor for subsequent demethylation and silencing. In *tet2*^*-/-*^*;tet3*^*-/-*^ mutants, 5mC is not converted to 5hmC, and the target locus (*nppa*) is ectopically expressed by retinal and brain cells. However, Tet-activity is also required for gene body 5hmC formation that is thought to serve as an active mark [30]. Thus, an alternative model can be envisioned wherein ectopic *nppa* expression also reflects indirect consequences of loss of tet2 and tet3 activity. In this model, an intermediate silencer/repressor gene is not properly expressed by *tet2*^*-/-*^*;tet3*^*-/-*^ mutant retinal cells due to the absence of activating 5hmC marks. This scenario is not unprecedented; Li et al recently demonstrated that *tet2*^*-/-*^*;tet3*^*-/-*^ mutants possess defects in hematopoiesis, but the mutants showed no changes in methylation or hydroxymethylation at key genes in the hematopoietic network; instead, in this context, Tet-mediated effects are likely the result of mis-regulation of the Notch pathway [27]. Similarly, mutation of the *de novo* DNA methyltransferase *dnmt3bb1* results in significantly altered expression of many hematopoietic and endothelial genes, although very few of these showed any changes in DNA methylation [91]. Therefore, genes identified as differentially expressed in *tet2*^*-/-*^*;tet3*^*-/-*^ mutants that displayed no changes in 5mC or 5hmC may not be direct targets of tet2 and tet3, but rather, reflect complex intrinsic or extrinsic regulatory pathways modulated by tet2 and tet3 activity. Testing this prediction will require genome-wide profiling of 5mC and 5hmC in the eye over multiple developmental time points and correlating these data with gene expression in a gene-by-gene fashion, as well as generating conditional loss of function tet2 and tet3 alleles such that their functions during later retinal development can be elucidated. CRISPR/Cas9 technology now makes this possible in zebrafish [92].

It is known that CXXC4/IDAX, a protein with homology to the tet3 N-terminal domain, functions as a direct inhibitor of Wnt signaling by competitively binding with Axin to Dvl [93]. In *tet2*^*-/-*^*;tet3*^*-/-*^ mutants, we detected no tet3 protein and 5hmC was almost completely absent from the genome, supporting a catalytic role for tet2 and tet3 during development. Significant changes in the expression of several Wnt ligands was detected in *tet2*^*-/-*^*;tet3*^*-/-*^ mutants, and these changes could result from this lack of catalytic activity (i.e. directly, from the lack of 5hmC formation at the loci, or indirectly, from the lack of 5hmC at loci encoding modulators of expression the Wnt ligands). However, alternatively, changes in Wnt ligand expression could also result from loss of the tet3 N-terminal CXXC domain, which functions independently of tet3 catalytic activity. Our data cannot yet differentiate between these possibilities. Importantly, this highlights the need to better understand the catalytic vs non-catalytic functions of tet proteins in specific tissues and organs, where tet proteins could regulate gene expression in several different ways.

Finally, in addition to DNA modifying enzymes like the Tets, chromatin regulators such as histone deacetylases and histone demethylases have also been shown to modulate cell extrinsic pathways during early retinal development [45, 94]. When combined with our work, these studies highlight that the epigenetic regulation of signaling events during development is likely to be a more significant and complex layer of regulation than previously realized. Deciphering this complex epigenetic regulation will require a comprehensive, genome-wide approach encompassing multiple profiling strategies (e.g. bisulfite sequencing, oxidative bisulfite sequencing, RNA-seq, ChIP-seq, and ATAC-Seq) in pure, isolated RPCs and differentiated retinal cell types from both wild-type embryos, as well as embryos deficient in key enzymes operating in these epigenetic pathways, like tet2 and tet3.

## Materials & methods

### Zebrafish lines and husbandry

Zebrafish were maintained at 28.5°C on a 14/10 light/dark cycle and treated in accordance with the University of Texas at Austin and University of Pittsburgh IACUC regulations governing animal research. Euthanasia utilized tricaine, following procedures standard in the field and as approved by the IACUC. Lines utilized in this study are: *tet2*^*au59*^, *tet3*^*au60*^, and *Tg(isl2b*:GFP*)*^*zc7*^ [55]. Embryos were incubated in the dark at 28.5°C and staged according to [95].

### Generation of tet mutants and overexpression lines

To generate the *tet2*^*-/-*^ and *tet3*^*-/-*^ mutant lines, TALEN constructs were generated using Golden Gate assembly [41, 96] targeting the following sequences (spacer in bold and restriction endonuclease recognition sites underlined): CATCCCAGATGGAAT**GGATAGA****TTTAAA****CTC**AACTTCTGCTTCAAC for *tet2*^*au59*^; GCTCTGGGAGATAAACT**GTAC****AGAGAAGTCAC**AGAAACCATCACCAAAT for *tet3*^*au60*^. Embryos at the 1-cell stage were injected with *in vitro* synthesized (Ambion) mRNA encoding the TALEN constructs (left and right arms) targeting *tet2* and *tet3*, separately, and raised to adulthood. At breeding age, potential founders were screened for germline transmission of mutations by sperm genomic PCR, followed by whole amplicon Sanger sequencing. Genotyping primers were: CACAAACCTCTCAGACAGGTCAGT (*tet2* forward), TCTCTGTTGACTTTCAGGGGCAG (*tet2* reverse), CAATGCCTAGATCAACCACTTAGTGTC (*tet3* forward), GTATCAGGAATGTGCAAACATCTCATTTG (*tet3* reverse). Founders with deletions that resulted in frameshifts and premature stop codons were outcrossed to wildtype females and embryos reared to adult. Potential heterozygotes were then screened for the desired mutation by restriction fragment length polymorphism (RFLP) using DraI for *tet2* and RsaI for *tet3*. RFLP fragments were resolved on a 1% agarose gel and mutant fragments were detected by the resistance to DraI and/or RsaI digestion (S2 Fig). To generate the double mutant line, heterozygotes carrying *tet2* or *tet3* mutations were crossed, and offspring were screened for the presence of both mutations by RFLP. *tet2*^*+/-*^*;tet3*^*+/-*^ fish were then incrossed to obtain *tet2*^*-/-*^*;tet3*^*-/-*^ embryos. Because homozygous mutation in either *tet2* or *tet3* alone does not affect viability, the remaining embryos survived to adult and a normal Mendelian distribution was obtained (S2 Fig).

### RT-PCR and Western blot analysis

RT-PCR for *tet2* and *tet3* was performed using exon-spanning primers listed in S3 Table. Embryos at 2dpf and 5dpf (n = 20 per genotype per condition) were euthanized and RNA extracted using RNeasy kit (Qiagen). cDNA libraries were generated using iScript cDNA synthesis kit (BioRad).

Western blot analyses was performed essentially as described [16] with slight modifications. At 3dpf, 40 embryos per condition were euthanized, de-yolked, and protein extracted. Samples were separated by electrophoresis on 4–12% bis-tris gel with NuPage MOPS SDS running buffer (Invitrogen) and transferred to PVDF membrane at 30V for 2 hours, then at 12V overnight at 4°C. Membranes were blocked in 1%BSA, 5% non-fat milk in TBST for 2 hours at RT and incubated in anti-TET3 rabbit polyclonal antibody (ab139311, Abcam) overnight at 4°C, then washed, incubated with HRP-conjugated donkey-anti-rabbit antibody (711-035-152, Jackson Immuno Research), rinsed, and incubated with substrate solution (Super Signal West Femto, Thermo Fisher). Images were acquired and band intensity quantified using ChemiDoc XRS+ system (BioRad). For normalization, membranes were stripped for 12 minutes in Restore Western Blot stripping buffer (Thermo Fisher), rinsed, re-blocked, probed with anti-actin mouse monoclonal antibody (CP01, Millipore) followed by HRP horse-anti-mouse secondary (7076, Cell Signaling) and imaged as above.

### In situ hybridization

Whole mount in situ hybridization experiments were performed essentially as described [97]. DIG-labeled RNA probes for *notch1a*, *deltaA*, *ascl1a*, *vsx2*, *pax6a*, *neurod4* and *atoh7* were described previously [43]. Probes for *tet1*, *tet2*, *tet3*, *lef1*, *wnt1*, *wnt9B*, *opn1sw1*, *opn1mw1*, and *nppa* were cloned from zebrafish cDNA using primers listed in S3 Table. PCR fragments were cloned into pGEM-T-Easy vector (Promega), sequence verified, linearized, and transcribed using SP6 and T7 polymerases with DIG RNA labeling mix (Roche). Synthesized RNA probes were purified using RNeasy kit (Qiagen), mixed 1:200 with hybridization buffer (50% formamide, 5xSSC, 0.1%tween, 5mg/ml yeast tRNA, 50μg/ml heparin), and heated to 68°C before use.

### Phylogenetic analysis

Amino acid sequences were downloaded from NCBI, using the following accession numbers: NP_085128.2 (human TET1), NP_001240786.1 (mouse Tet1), AHE93329.1 (zebrafish tet1), NP_001120680.1 (human TET2), NP_001035490.2 (mouse Tet2), AHE93330.1 (zebrafish tet2), NP_001274420.1 (human TET3), NP_898961.2 (mouse Tet3), AHE93331.1 (zebrafish tet3). Alignments and phylogenetic trees were constructed using Geneious Tree Builder software with standard neighbor-joining method (Biomatters).

### BrdU incorporation and PLM assays

5-bromo-2-deoxyuridine (BrdU) incorporation was performed using a 15-minute pulse for PLM assays, and a 2-hour time window for 48hpf-5dpf assays. Embryos were treated in 0.3% BrdU, fixed in 4% PFA in PBS, embedded, and cryosectioned at 12μm. Sections were treated with 4M HCl at 37°C for 10min, blocked in block solution (5% normal goat serum, 0.1% tween, 1% DMSO, in PBS), incubated with anti-BrdU (1:250; Abcam) in block overnight at 4°C, stained with anti-rat Cy3 secondary (1:250) and counterstained with Sytox green at 1:10,000 (Molecular Probes). Cells undergoing mitosis were detected using anti-phospho histone H3 (ser10) (1:250) (Millipore 06–570), stained with anti-rabbit Cy2 secondary (1:250), and counterstained with DAPI (1:500).

### TUNEL assay

Embryos at 36hpf, 3dpf, 4dpf, and 5dpf were fixed in 4% PFA 1xPBS at 4°C overnight, cryosectioned at 12μm, and processed for TUNEL using TMR Red in situ cell death detection kit (Roche) per manufacturer’s protocol.

### Immunohistological analysis

Immunohistochemistry was perform as described [98], with the following antibodies: zpr-1 (cones; ZIRC), zpr-3 (rods; ZIRC), zrf-1/gfap (Muller glia cells; ZIRC), Zn8 (ganglion cells; ZIRC), and HuC/D (ganglion and amacrine cells; Molecular Probes). Embryos were cryosectioned at 12μm and incubated with primary antibodies diluted at 1:200 in block overnight at 4°C, then incubated with secondary antibody (anti-mouse Cy3) for 2hrs. Sections were counterstained with Alexa Fluor-633 Phalloidin at 1:100 and Sytox green at 1:10,000 (Molecular Probes) or mounted using Vectashield with DAPI (Vector Labs). Images were acquired using Zeiss LSM5 and/or Olympus FV1200 confocal microscopes, and analyzed using ImageJ with Cell Counter plug-in (imagej.nih.gov).

### Whole-mount chromogenic immunostaining

Embryos were fixed in 4% PFA 1x PBS at 4°C overnight, rinsed once in PBST (0.1% tween-20, 1xPBS), once in water, and treated with 100% acetone for 7min at -20°C to permeabilize the tissue, then rinsed one time each in water, PBST and PBDTX (1%BSA, 1%DMSO, 0.1% TritonX, 1xPBS, pH = 7.3). Embryos were blocked for 1hr (2%NGS in PBDTX), incubated in Zn8 primary antibody (ZIRC) at 1:200 dilution 4°C overnight, washed 4 x 20min in PBDTX, and incubated in secondary horse anti-mouse HRP-tagged secondary (Cell Signaling) at 1:1,000 dilution for 2hrs. Embryos were then washed in PBSTX (0.5% Triton-X, 1xPBS) 4 x 20min, incubated in DAB working solution (Vector Labs) for 2-10min until staining was visible, rinsed in water, and stored in PBS before imaging.

### RNA extraction and transcriptome analysis

One hundred zebrafish eyes were dissected at either 36hpf or 72hpf using a flame-sharped tungsten wire, and RNA extracted using Qiagen RNeasy kit as described [67]. For 36hpf, library preparation with polyA mRNA capture and sequencing was performed using Illumina NextSeq 500 paired-end 75bp reads. 450 million reads were generated. Raw FASTQ sequences were quality checked, trimmed, and mapped using CLC Genomic Workbench 9.0.1 to zebrafish reference genome GRCz10 at 85% mapping efficiency. Transcript abundances were calculated and differentially expressed genes (DEGs) were identified using CLC Genomic Workbench 9.0.1. For 72hpf, library was prepared as above and sequenced on an Illumina HiSeq 2500 PE2x125. 66 million reads were generated. FASTQ sequences were quality checked using FastQC (Babraham Bioinformatics), mapped to GRCz10 using TopHat, and DEGs were identified using Cufflinks package from Tuxedo suite [72]. Genes with expression values above log2 fold-change of 2 are considered differentially expressed. All computational analyses utilized the Texas Advanced Computing Center and University of Pittsburgh Center for Simulation and Modeling. Raw and processed data are publicly available through NCBI Gene Expression Omnibus (accession number GSE80134).

Functional annotation was done using DAVID Bioinformatics 6.8 (https://david.ncifcrf.gov). Differentially expressed gene lists from RNA-seq were filtered for log2 fold-change of 2 or higher and submitted to DAVID Gene Ontology for biological pathways (GOTERM_BP_DIRECT).

### Mosaic retinal analysis

Shield-stage transplantation experiments were performed essentially as described [63]. Embryos were injected with Alexa Fluor 488 dextran (10,000 MW, anionic, fixable) diluted at 1% in 0.2M KCl. Cells were transplanted from labeled donor embryos into unlabeled host embryos at the shield stage, targeting the presumptive retinal field [64]. Approximately 10 cells were transplanted per host embryo to minimize the ‘community’ effect resulting from clones that are too large. Embryos were sorted at 24hpf for donor clone contribution, and fixed at 72hpf for sectioning and immunohistochemistry.

### 5mC and 5hmC quantification

Bisulfite sequencing was performed using EZ DNA Methylation-Direct kit (Zymo Research). Eye tissues at 72hpf were dissected (n = 7 per condition) and immediately processed through proteinase K digestion and bisulfite conversion. Converted DNA was purified and amplified using hot-start ZymoTaq and bisulfite-specific primer pairs (S3 Table). PCR amplicons (~300bp) were either directly sequenced or sub-cloned for sequencing. Sequencing traces were analyzed using QUMA (RIKEN, Japan). Our bisulfite treatment procedure generally yielded ~98% conversion. Clones that contained low quality sequences were manually excluded from the analysis.

Locus-specific 5hmC quantification was performed using the Quest 5hmC Detection kit (Zymo Research). Briefly, genomic DNA was extracted at 72hpf using Purelink Genomic DNA purification kit (Invitrogen). Genomic DNA was divided into three groups: 1) Glycosylated and digested with a glucosyl-5hmC sensitive endonuclease, MspI [+GT]; 2) Unglucosylated and digested with MspI [-GT] (negative control); 3) unprocessed genomic DNA [untreated] (positive control). All DNA samples were purified, and equal amounts used as templates for quantitative real-time PCR. Quantitative real-time PCR was done using SYBR green master mix in 10ul volume, and reactions run in a CFX384 detection system (BioRad). Cq values were first evaluated by comparing the difference between the +GT and -GT, and between +GT and untreated. If Cq_+GT_ is close to (<1 Cq difference) Cq_untreated_, the locus is considered fully hydroxymethylated. Conversely, if Cq_+GT_ is close to Cq_-GT_, the locus is considered non-hydroxymethylated. For each locus with Cq values that pass these criteria, they are considered partially hydroxymethylated, and percent 5hmC was calculated as follows: %5hmC = {[Cq_-GT_−Cq_+GT_] / [Cq_-GT_−Cq_untreated_]} *100.

### Enzyme-linked immunosorbent assay (ELISA)

Sandwich-based 5hmC ELISA was performed according to the manufacturer’s protocol (Zymo Research). Genomic DNA samples were extracted at 5dpf using Purelink Genomic DNA extraction kit (Invitrogen), and diluted to 1ng/μl in water, denatured by heating at 98°C and cooling on ice, and 5hmC DNA was bound to the ELISA plate coated with anti-5hmC polyclonal antibody (1:1,000). Bound DNA was detected with anti-DNA HRP antibody (1:100), and was allowed to develop for 20min. 410nm absorbance was measured by a plate reader (BioTek), and a standard curve generated using linear regression from five DNA samples with known concentrations of 5hmC. Percent 5hmC was calculated as follows: %5hmC = (absorbance—y-intercept)/slope. Note that percent 5hmC in ELISA is based on the total number of hydroxymethylated cytosines, calibrated to standards (set of DNA with known 5hmC%). For example, 0.1% 5hmC means 1 of every 1,000 cytosines is 5hmC. Percent 5hmC in the site-specific glucosylation/digestion (Quest) assay represents the relative amount of ‘protected’ 5hmC at each MspI (CCGG) site analyzed, compared to the two internal controls for each locus: fully digested DNA (representing 0% 5hmC) and undigested DNA (representing 100% 5hmC). Thus, these two numbers are not directly comparable, but should be in agreement with each other.

### Pharmacological treatment

*tet2*^*-/-*^*;tet3*^*-/-*^ mutants and sibling embryos carrying *isl2b*:GFP transgene were dechorionated and incubated from 24hpf to 72hpf in embryo medium with 50μM DAPT (N-[N-(3,5-difluorophenacetyl)-L-alanyl]- S-phenylglycine-t-butyl ester; InSolution γ-secretase inhibitor IX, 565784, Calbiochem), 5μM IWR-1-endo (5.04462.0001, Calbiochem), or 1% DMSO as vehicle control. For BIO treatment, wildtype embryos carrying *isl2b*:GFP transgene were incubated in 2μM BIO (2’Z,3’E-6-Bromoindirubin-3’-oxime, B1686-5MG, Sigma-Aldrich) from 24-72hpf. All embryos were fixed at 72hpf in 4%PFA 1xPBS, sectioned, and processed for immunostaining. Optic nerve diameter measurements were done in 5–7 embryos per condition at optic nerve head, using FluoView software (Olympus). P-values were calculated using two-way ANOVA with multiple comparison (for DAPT and IWR) and two-tailed unpaired t-test (for BIO) using Prism GraphPad.

## Supporting information

S1 Fig Tet-family gene expression and phylogenetic analyses. (A) An unrooted phylogenetic tree constructed from mouse, human and zebrafish Tet1, 2 and 3 proteins. (B-D) *tet1*, *tet2* and *tet3* are ubiquitously expressed at 24hpf. At 48 (E-G) and 72hpf (H-J) *tet2* and *tet3* are expressed in the inner nuclear layer (INL; arrowhead) and ganglion cell layer (GCL; arrows), and faintly in the outer nuclear layer (ONL). n>8 per gene per time point. Scale bar = 20μm.
(TIF)

S2 Fig *tet2* and *tet3* RFLP genotyping and genotypic distribution in adults. **(A)** Mutations were detected by restriction fragment length polymorphism (RFLP). Mutant alleles lack the recognition site for DraI (for *tet2*) and RsaI (for *tet3*), and are therefore undigested. (B) At 3-months, 96 fish were individually genotyped by RFLP. The genotypic distribution follows a Mendelian distribution for a dihybrid cross, except for the absence of *tet2*^*-/-*^*;tet3*^*-/-*^ mutations, which are embryonic lethal and *tet2*^*+/-*^*;tet3*^*-/-*^ which are juvenile lethal.
(TIF)

S3 Fig *tet2* transcript and tet3 protein are undetectable in *tet2*^*-/-*^*;tet3*^*-/-*^ mutants. (A) At 2dpf and 5dpf, *tet2* transcripts are present in sibling but undetectable by RT-PCR in *tet2*^*-/-*^*;tet3*^*-/-*^ mutants indicating degradation, presumably via nonsense-mediated decay. (B) *tet3* transcripts are present in both sibling and *tet2*^*-/-*^*;tet3*^*-/-*^ at both time points. N = 20 embryos per condition, and experiments done in biological triplicates. RT-PCRs for *tet2* and *tet3* were done in parallel from the same cDNA pools. (C,D) At 3dpf, tet3 protein (225 kDa) is absent from *tet2*^*-/-*^*;tet3*^*-/-*^ mutants. N = 40 embryos per condition, and experiments done in biological triplicates. P<0.0001, unpaired t-test.
(TIF)

S4 Fig *tet2*^*-/-*^*;tet3*^*-/-*^ embryos possesses few apoptotic cells prior to 3dpf. TUNEL labeling was performed on cryosections of *tet2*^*-/-*^*;tet3*^*-/-*^ and sibling embryos at 36hpf, 3dpf, 4dpf, and 5dpf. No difference was observed at 36hpf (A,E), and few apoptotic cells are observed in *tet2*^*-/-*^*;tet3*^*-/-*^ at 3dpf (B,F; arrows). More apoptotic cells are observed in *tet2*^*-/-*^*;tet3*^*-/-*^ at 4dpf and 5dpf (C-D; G-H). Images are representatives of at least n = 3 embryos examined. DNA (blue), TUNEL signal (red).
(TIF)

S5 Fig *tet2*^*-/-*^*;tet3*^*-/-*^ embryos possesses fewer amacrine cells at 3dpf. Number of HuC/D-positive neurons in the INL (amacrine cells) is significantly lower in *tet2*^*-/-*^*;tet3*^*-/-*^ eyes than in sibling, although the number of HuC/D-positive cells in the GCL (consisting of ganglion and displaced amacrine cells) is not significantly different. Error bars = ± 1 S.D. Significance cut-off for p-value = 0.05 (two-tailed, unpaired t-test).
(TIF)

S1 Table List of genes differentially expressed in *tet2*^*-/-*^*;tet3*^*-/-*^ eyes at 36hpf when compared to phenotypically wild-type siblings. Expression values are cutoff at log2 fold-change over 2 or under -2.
(XLSX)

S2 Table List of genes differentially expressed in *tet2*^*-/-*^*;tet3*^*-/-*^ eyes at 72hpf when compared to phenotypically wild-type siblings. Expression values are cutoff at log2 fold-change over 2 or under -2.
(XLSX)

S3 Table List of primers used for bisulfite sequencing, Quest 5hmC qPCR, and in situ probe cloning. (XLSX)

S4 Table Methylation status and 5hmC enrichment at candidate loci. (XLSX)
