# Supplementary figures and images for: Tet-mediated DNA hydroxymethylation regulates retinal neurogenesis by modulating cell-extrinsic signaling pathways
Source: PLoS Genet. 2017 Sep 19;13(9):e1006987. doi: 10.1371/journal.pgen.1006987 (PMC5621703; doi:10.1371/journal.pgen.1006987)

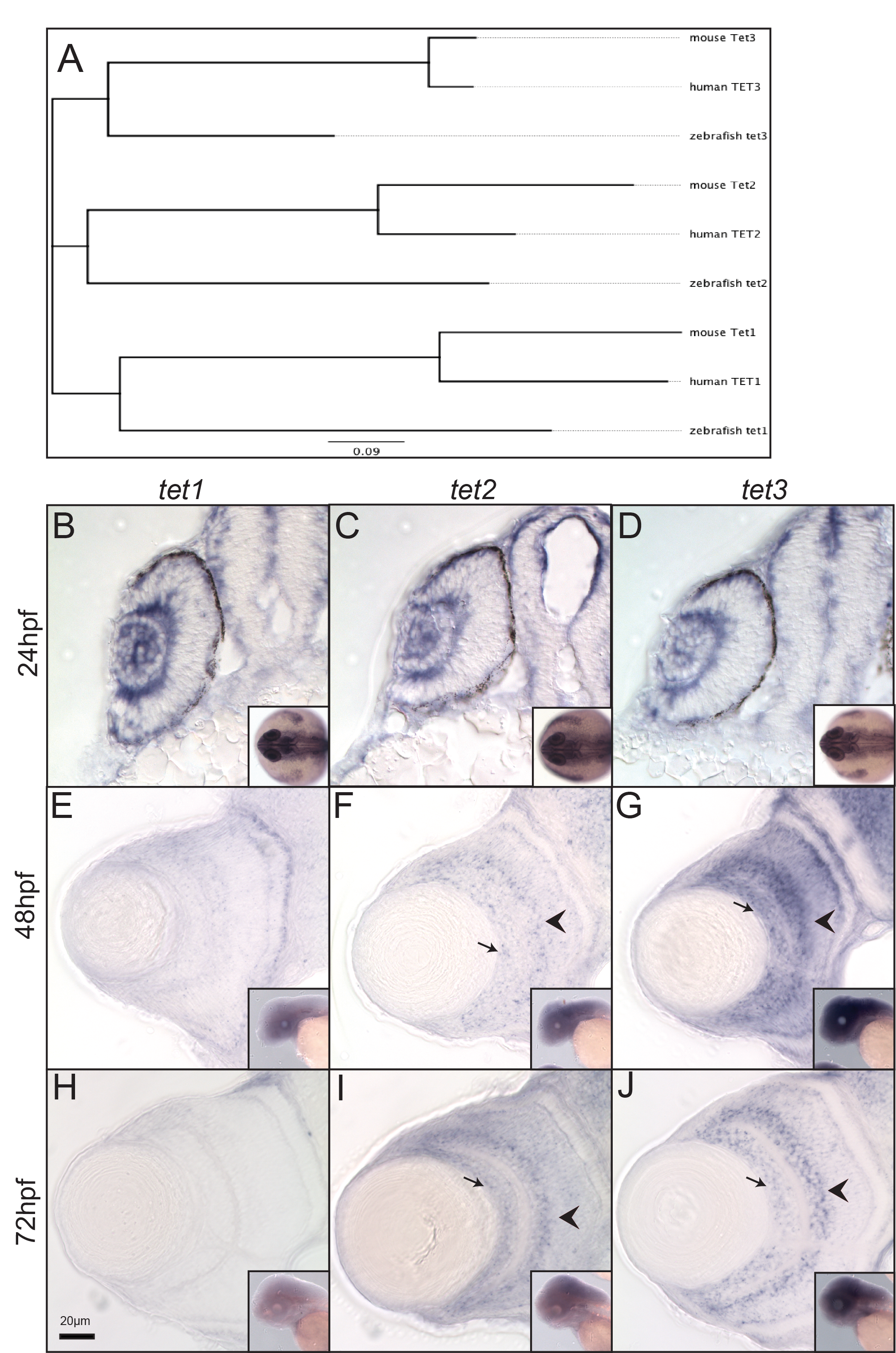

Supplement: S1 Fig — (A) An unrooted phylogenetic tree constructed from mouse, human and zebrafish Tet1, 2 and 3 proteins. (B-D) tet1, tet2 and tet3 are ubiquitously expressed at 24hpf. At 48 (E-G) and 72hpf (H-J) tet2 and tet3 are expressed in the inner nuclear layer (INL; arrowhead) and ganglion cell layer (GCL; arrows), and faintly in the outer nuclear layer (ONL). n>8 per gene per time point. Scale bar = 20μm. (TIF) [file pgen.1006987.s001.tif]

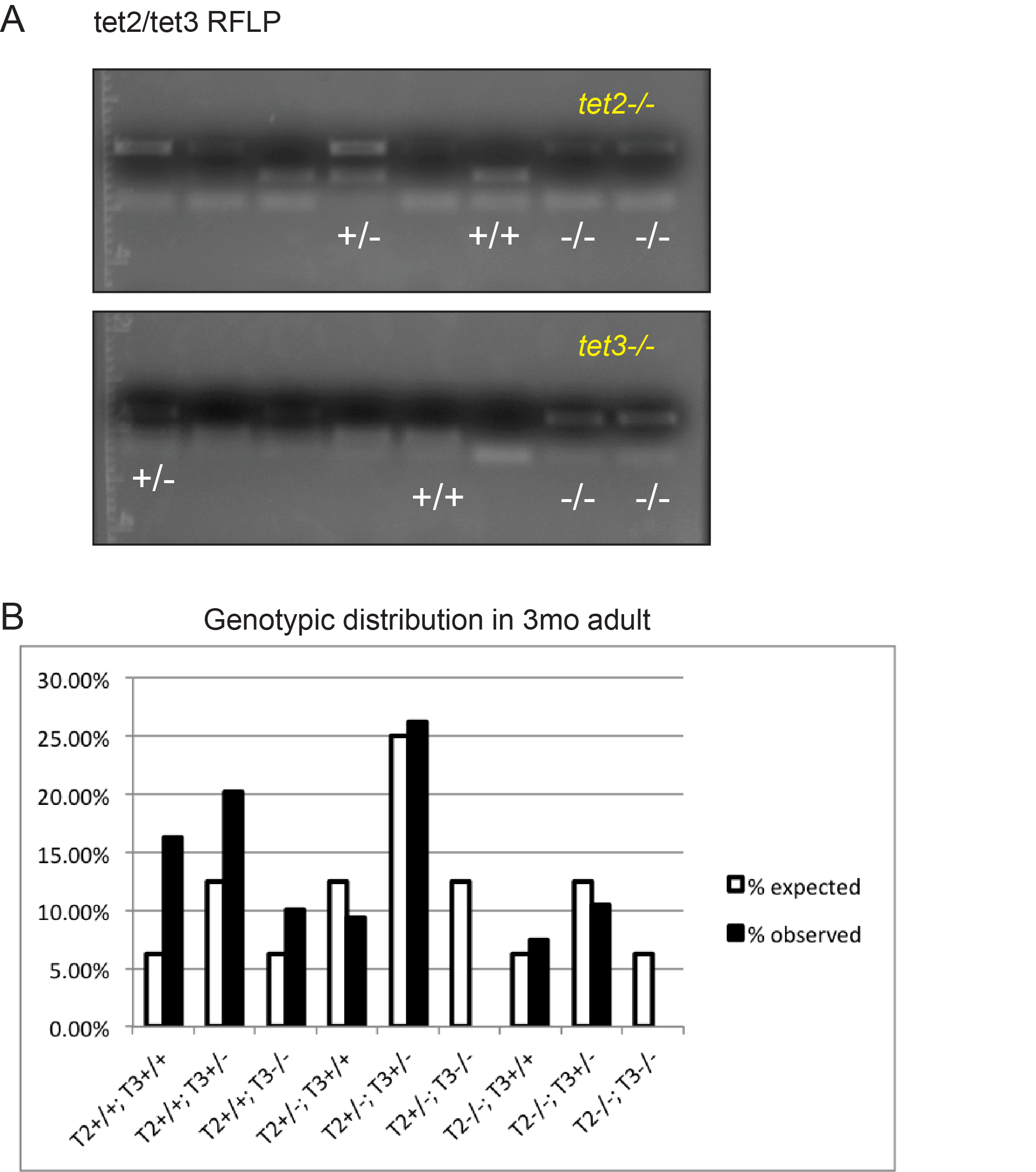

Supplement: S2 Fig — (A) Mutations were detected by restriction fragment length polymorphism (RFLP). Mutant alleles lack the recognition site for DraI (for tet2) and RsaI (for tet3), and are therefore undigested. (B) At 3-months, 96 fish were individually genotyped by RFLP. The genotypic distribution follows a Mendelian distribution for a dihybrid cross, except for the absence of tet2-/-;tet3-/- mutations, which are embryonic lethal and tet2+/-;tet3-/- which are juvenile lethal. (TIF) [file pgen.1006987.s002.tif]

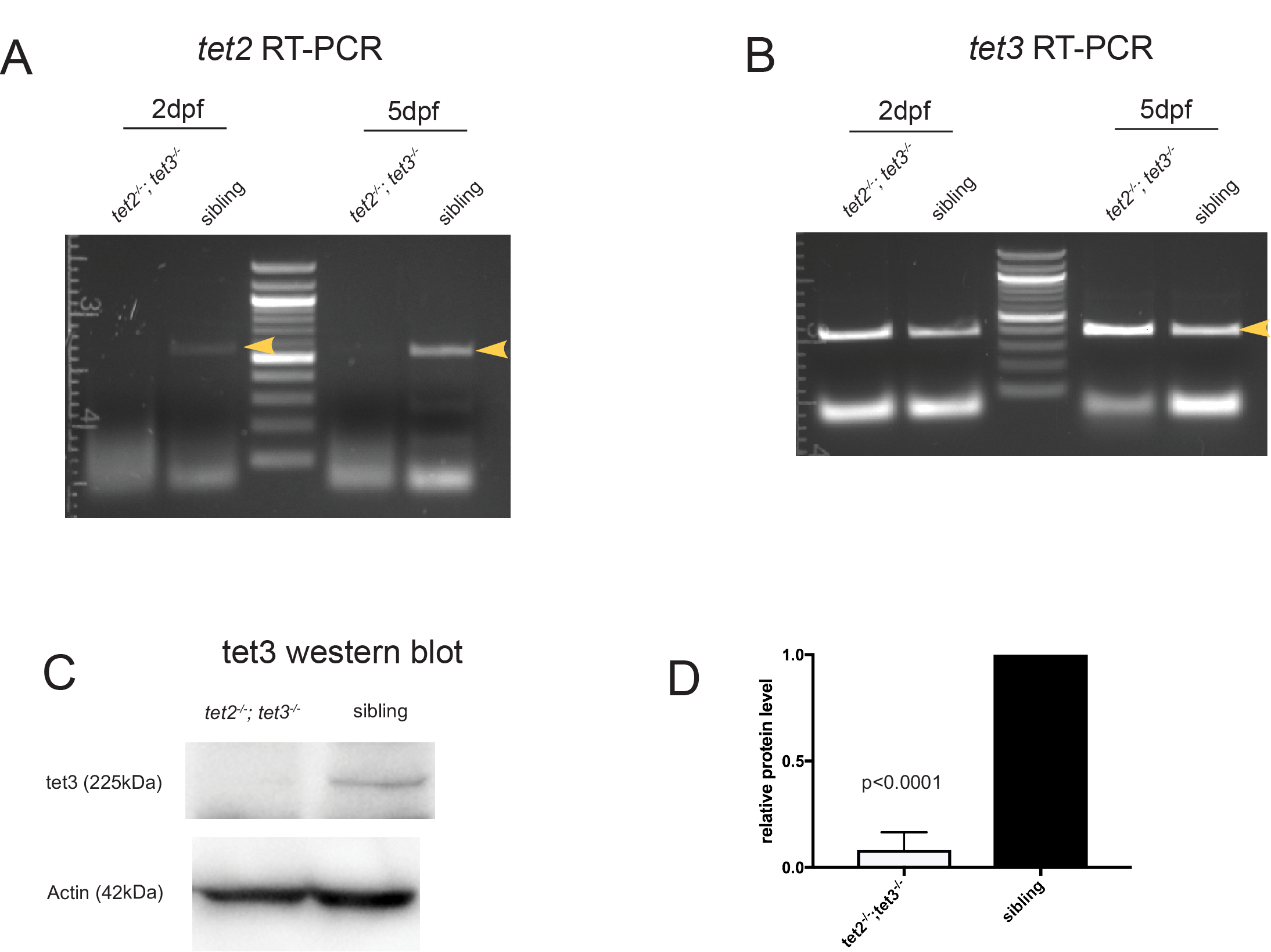

Supplement: S3 Fig — (A) At 2dpf and 5dpf, tet2 transcripts are present in sibling but undetectable by RT-PCR in tet2-/-;tet3-/- mutants indicating degradation, presumably via nonsense-mediated decay. (B) tet3 transcripts are present in both sibling and tet2-/-;tet3-/- at both time points. N = 20 embryos per condition, and experiments done in biological triplicates. RT-PCRs for tet2 and tet3 were done in parallel from the same cDNA pools. (C,D) At 3dpf, tet3 protein (225 kDa) is absent from tet2-/-;tet3-/- mutants. N = 40 embryos per condition, and experiments done in biological triplicates. P<0.0001, unpaired t-test. (TIF) [file pgen.1006987.s003.tif]

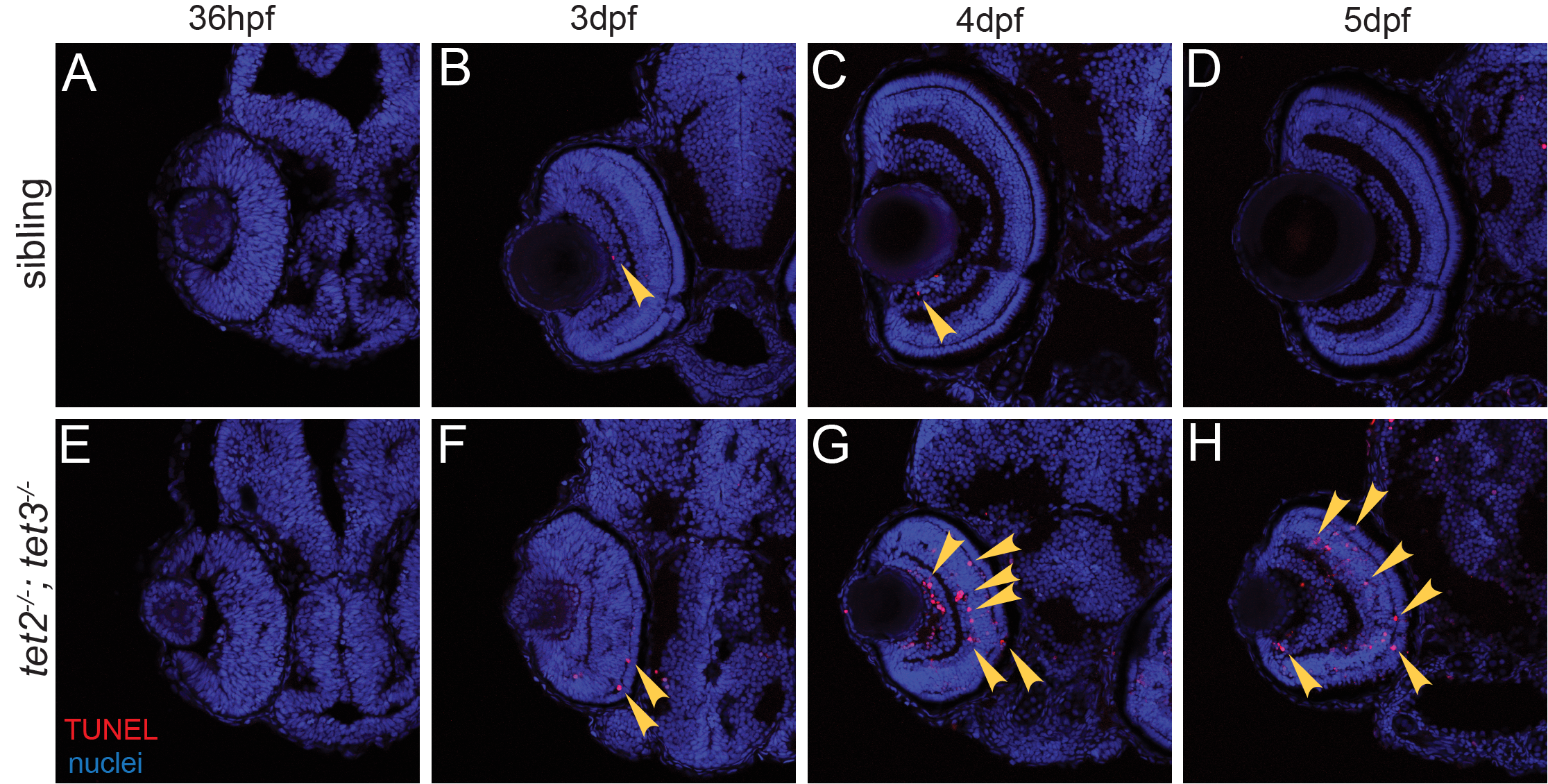

Supplement: S4 Fig — TUNEL labeling was performed on cryosections of tet2-/-;tet3-/- and sibling embryos at 36hpf, 3dpf, 4dpf, and 5dpf. No difference was observed at 36hpf (A,E), and few apoptotic cells are observed in tet2-/-;tet3-/- at 3dpf (B,F; arrows). More apoptotic cells are observed in tet2-/-;tet3-/- at 4dpf and 5dpf (C-D; G-H). Images are representatives of at least n = 3 embryos examined. DNA (blue), TUNEL signal (red). (TIF) [file pgen.1006987.s004.tif]

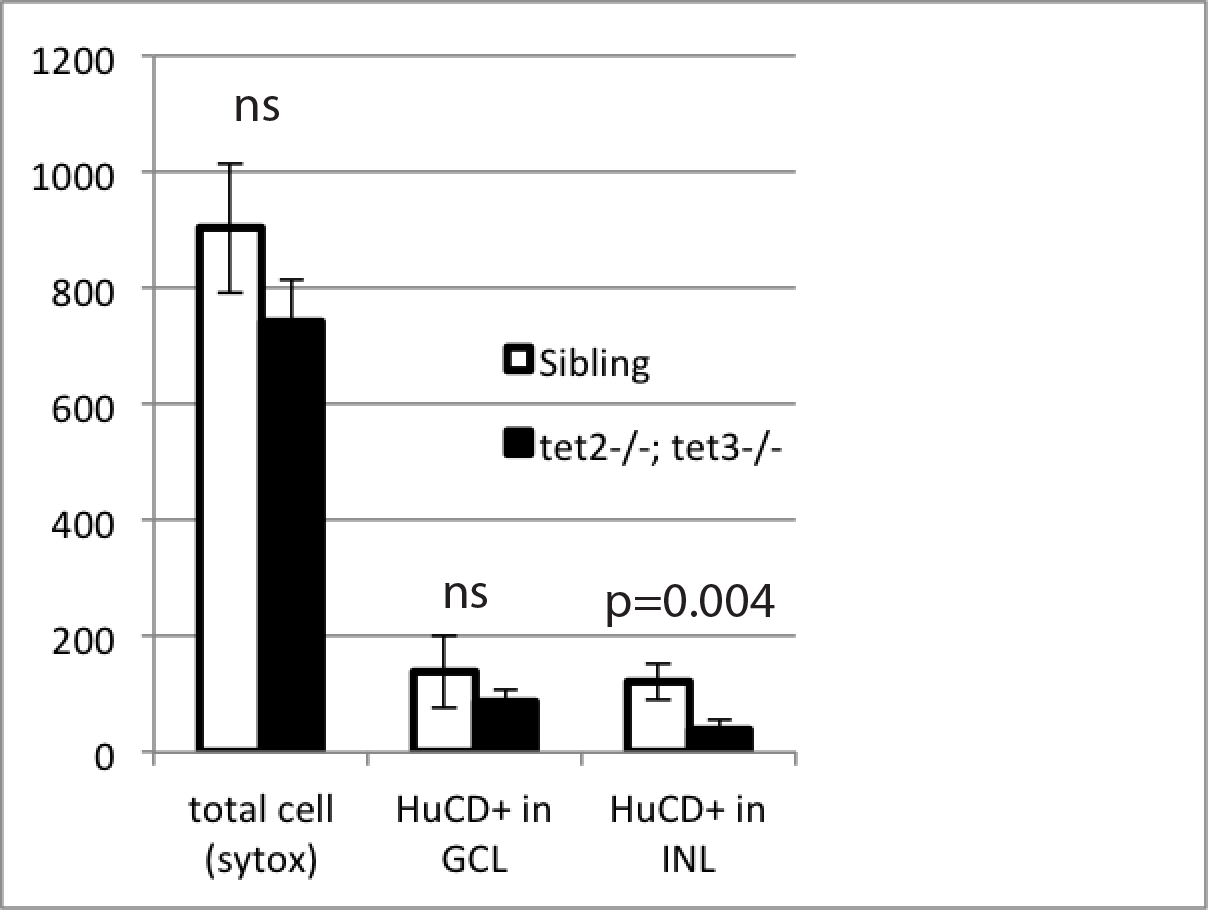

Supplement: S5 Fig — Number of HuC/D-positive neurons in the INL (amacrine cells) is significantly lower in tet2-/-;tet3-/- eyes than in sibling, although the number of HuC/D-positive cells in the GCL (consisting of ganglion and displaced amacrine cells) is not significantly different. Error bars = ± 1 S.D. Significance cut-off for p-value = 0.05 (two-tailed, unpaired t-test). (TIF) [file pgen.1006987.s005.tif]
